# Ovarian Aging: Mechanisms, Age‐Related Disorders, and Therapeutic Interventions

**DOI:** 10.1002/mco2.70481

**Published:** 2025-11-16

**Authors:** Xingyu Liu, Yuanqu Zhao, Yanzhi Feng, Shixuan Wang, Jinjin Zhang

**Affiliations:** ^1^ Department of Obstetrics and Gynecology Tongji Hospital, Tongji Medical College, Huazhong University of Science and Technology Wuhan China; ^2^ National Clinical Research Center for Obstetrical and Gynecological Diseases Huazhong University of Science and Technology Wuhan China; ^3^ Key Laboratory of Cancer Invasion and Metastasis Ministry of Education, Huazhong University of Science and Technology Wuhan China

**Keywords:** age‐related diseases, mechanism, ovarian aging, therapeutic interventions

## Abstract

Ovarian aging is a fundamental process in female reproductive biology with broad implications for overall health and aging. As global populations age, understanding its mechanisms and systemic effects has gained urgent clinical relevance. The ovary, beyond its reproductive role, is increasingly recognized as a regulator of systemic aging due to the widespread presence of estrogen receptors. Declining ovarian function accelerates not only reproductive senescence but also contributes to age‐related disorders including osteoporosis, neurodegenerative diseases, and cardiovascular conditions. However, research on ovarian aging remains fragmented, lacking integrative analysis. This review synthesizes recent advances in the cellular and molecular mechanisms underpinning ovarian aging, such as genomic instability, metabolic and oxidative stress, and microenvironmental alterations. It further discusses how ovarian decline influences systemic aging pathways and disease susceptibility and evaluates emerging therapeutic strategies such as antioxidant interventions, stem cell therapy, and ovarian tissue transplantation. By providing a comprehensive overview of ovarian aging from mechanisms to interventions, this review aims to bridge existing knowledge gaps and inspire future research toward improving women's healthspan and quality of life.

## Graphical abstract

A comprehensive overview of the molecular mechanisms underlying ovarian aging, its association with age‐related diseases, and potential therapeutic interventions. Ovarian aging involves DNA damage, mitochondrial dysfunction, oxidative stress, telomere attrition, and follicular depletion, driving age‐related infertility, menopause, and multiple systemic disorders. Emerging therapeutic interventions include antiaging compounds, stem cells therapy, ovarian tissue transplantation, as well as innovative modalities such as tissue engineering and bioactive materials, drug delivery to delay ovarian senescence and associated pathologies.

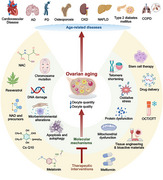

## 1 Introduction

The ovary is the central reproductive and endocrine organ in women, responsible for producing fertilizable oocytes and secreting steroid hormones that regulate systemic physiological processes. It is among the first organs to exhibit signs of aging, with a noticeable decline in fertility beginning in the late 20s and accelerating after the age of 35 years [1]. Menopause, typically occurring around the age of 51 years [1], marks the near‐complete cessation of ovarian function and a sharp drop in estrogen levels [2]. This accelerated decline contrasts with increasing human lifespan, making ovarian aging a critical determinant of women's healthspan and quality of life.

Traditionally, the physiological consequences of ovarian aging have been largely ascribed to the decline in estrogen levels. Estrogen plays a critical role in numerous systemic processes, including the maintenance of cardiovascular health, bone mineral density, immune regulation, and cognitive function [3]. Accordingly, the postmenopausal reduction in estrogen is associated with an increased risk of various age‐related diseases, such as osteoporosis, cardiovascular disease (CVD), and cognitive impairment, attributable to the extensive distribution of estrogen receptors (ERs) across diverse tissues and organ systems [4]. Beyond these well‐characterized effects, emerging evidence implicates ovarian aging in the pathogenesis of skin aging [5], chronic kidney disease (CKD) [6], type 2 diabetes [7], chronic obstructive pulmonary disease (COPD) [8], and even reduced overall life expectancy [9]. Thus, ovarian aging not only triggers estrogen deficiency but also initiates a cascade of systemic dysfunctions that promote multisystem aging and disease progression.

A growing body of evidence highlights the importance of nonestrogenic mechanisms by which ovarian aging contributes to systemic pathophysiology. With advancing age, ovarian cells undergo cellular senescence and acquire a senescence‐associated secretory phenotype (SASP), characterized by the release of proinflammatory cytokines (e.g., IL‐1α, IL‐6), chemokines, and matrix metalloproteinases. These factors may enter the circulation, driving chronic low‐grade inflammation (or “inflammaging”) that promotes tissue damage across vascular, skeletal, and neural systems, thereby accelerating the development of CVD, osteoporosis, and neurodegeneration [10, 11, 12]. Beyond inflammatory mediators, other ovarian‐derived factors influence systemic aging trajectories. For instance, declining levels of anti‐Müllerian hormone (AMH), a key biomarker of ovarian reserve, have been linked in emerging clinical studies to increased cardiovascular risk [13, 14] and insulin resistance [15]. Collectively, these findings reframe ovarian aging not merely as a reproductive milestone, but as a central endocrine and inflammatory driver with profound implications for multisystem aging and disease.

Against this backdrop, this review synthesizes emerging evidence supporting the premise that ovarian aging functions as a critical endocrine driver of multiorgan aging and represents a promising therapeutic target for mitigating age‐related diseases. Given the growing emphasis on aging biology and precision medicine, deciphering the molecular and cellular processes underlying ovarian aging has become increasingly urgent. Recent studies have delineated multiple determinants of ovarian decline, including genetic mutations [16, 17], chromosomal cohesion defects [16], telomere shortening [16], mitochondrial dysfunction [18], reactive oxygen species (ROS) accumulation [18], and altered ovarian microenvironments [18]. These advances not only deepen our mechanistic insight but also provide a foundation for developing targeted interventions to delay ovarian aging and its systemic consequences.

This review is structured to provide a systematic and integrated examination of ovarian aging and its systemic implications. We begin by examining the quantitative depletion and qualitative deterioration of oocyte throughout the ovarian aging process. Then, we delve into the core molecular and cellular mechanisms driving ovarian aging, encompassing genomic instability, metabolic and oxidative stress (OS), and alterations in the tissue microenvironment. We then discuss the systemic effects of declining estrogen levels following ovarian aging, with a focus on its impact on cardiovascular, skeletal, neurocognitive, metabolic health, among other systems. Subsequently, we summarize current and emerging biomarkers and diagnostic tools for assessing ovarian aging. Finally, we evaluate novel therapeutic strategies, such as antiaging drugs, stem cell‐based therapies, ovarian transplantation, and tissue engineering, emphasizing their mechanisms, efficacy, and translational potential. We propose that ovarian aging may act as a key catalyst for age‐related diseases in women. Through this organized framework, the review advances a unifying perspective: ovarian aging serves as a critical regulator of multisystem aging and a pivotal target for interventions aimed at promoting healthy longevity.

## 2 Ovarian Aging Hallmarks‐Quantitative Depletion and Qualitative Deterioration of Oocyte

Female reproductive potential is determined by both the quantity and quality of oocytes, both of which exhibit significant age‐related declines. The ovarian reserve steadily diminishes from fetal life onward, while the developmental competence of oocytes shows a more pronounced deterioration after the early thirties. These concurrent processes define the natural timeline of female fertility and present major challenges in clinical reproductive medicine.

### 2.1 Oocyte Quantity

**FIGURE 1:**
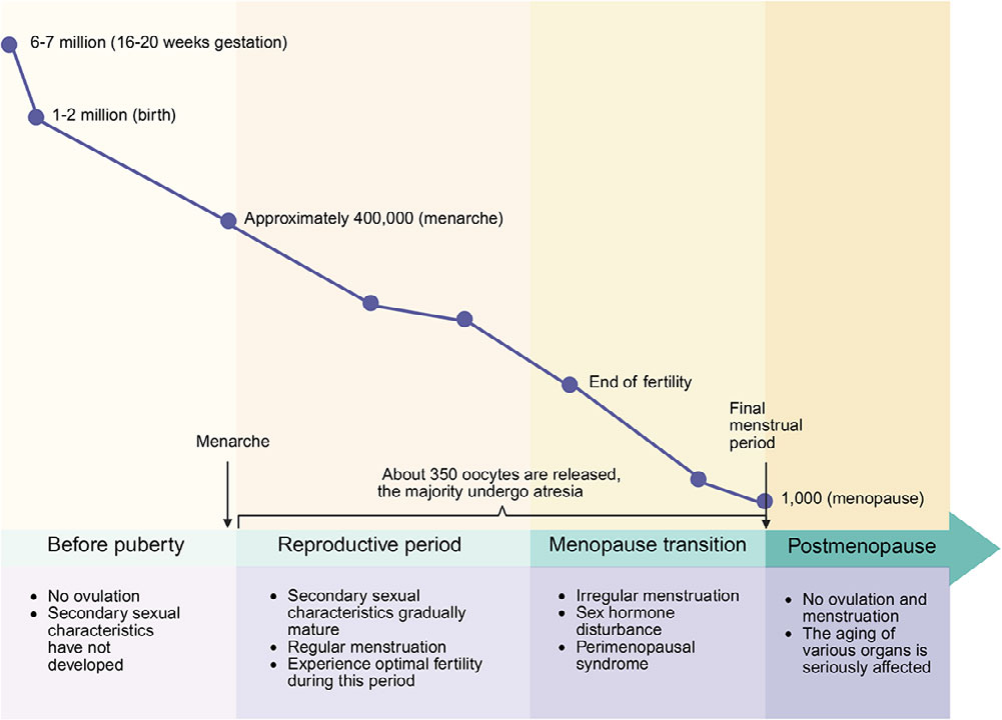
The life stages of the ovary. Diagram illustrating ovarian changes across different life stages, from before puberty to postmenopause, showing the dynamic alterations in follicle number and ovarian function. Created with BioRender.com.

Women are born with a finite pool of oocytes, the foundation of their reproductive potential. By the fifth week of gestation, the female fetal ovary contains approximately 500 to 1300 primordial germ cells (PGCs). This population expands rapidly, reaching a peak of nearly 7 million oocytes by 16–20 weeks of gestation, each encapsulated by granulosa cells (GCs) to form primordial follicles (Figure 1). However, the ovarian reserve undergoes a progressive and irreversible decline. At birth, only 1–2 million primordial follicles remain, a number that further diminishes to approximately 400,000 by puberty. Over the course of reproductive life, only about 350 oocytes are selected for ovulation, while the majority undergo atresia. Mathematical modeling indicates that the depletion of ovarian reserve follows a nonlinear trajectory, with a marked acceleration around the age of 30 years, primarily driven by increased oocyte atresia. By the time of menopause, typically occurring at an average age of 51 years, fewer than 1000 primordial follicles persist [18].

### 2.2 Oocyte Quality

In addition to the follicular number diminishing, oocyte quality also deteriorates with advancing maternal age. This decline becomes apparent after the age of 31 years, coinciding with a reduction in the ovarian reserve. The progressive deterioration of oocyte quality contributes not only to diminished fertility prior to menopause but also to endocrine dysregulation in reproductively active older women. At the cellular level, oocyte senescence involves inefficient cross maturation, meiotic spindle disruption, telomere shortening, accumulation of DNA damage, and mitochondrial dysfunction [1]. The decreased fertility associated with aging primarily arises from increased meiotic nondisjunction during oogenesis, leading to aneuploidy and a higher risk of unsuccessful pregnancies [19].

Current research on declining oocyte quality has largely focused on cytological mechanisms, particularly DNA damage and mitochondrial dysfunction. However, this approach neglects the functional dimension of oocyte aging, including reduced developmental capacity, impaired blastocyst formation, and dysregulated gene expression. Accumulation of mitochondrial DNA (mtDNA) mutations with age compromise energy homeostasis, spindle integrity, and increase aneuploidy and developmental abnormalities [20, 21].

Traditional assisted reproductive technologies (ARTs) oocyte assessments based on morphology (zona pellucida thickness, polar body morphology, cytoplasmic uniformity) have limited predictive power. Newer functional and subcellular parameters, like cytoplasmic granularity, mitochondrial membrane potential (Δ*Ψ*_m_), and chromatin structure, offer more accurate insights. The ESHRE–ALPHA Istanbul Consensus reveals that excessive cytoplasmic granulation or very large vacuoles (>25 µm) may be morphologic features associated with lower developmental competence [22]. ∆*Ψ*_m_, reflecting the metabolic activity of oocytes, increases during maturation and exhibits localized enrichment near the meiotic spindle, correlating strongly with blastocyst formation potential [23]. Additionally, chromatin configuration in germinal vesicle (GV) oocytes, classified into nonsurrounded nucleolus and surrounded nucleolus (SN) types, serves as a predictive marker of nuclear synchrony and oocyte competence, with SN oocytes typically demonstrating superior maturation and developmental outcomes [24]. Combining morphological and functional indicators enhances oocyte assessment for improved embryo selection and personalized ARTs.

Emerging and established markers of oocyte quality show variable but quantifiable links with ART outcomes (Table S1). While zona pellucida thickness offers limited predictive value [25, 26], intact first polar bodies consistently associate with higher implantation and pregnancy rates [27]. Cytoplasmic uniformity, though heterogeneously defined across studies, has been linked to improved implantation [28]. Cytoplasmic dysmorphisms—particularly refractile bodies and central granularity—are reliable indicators of reduced developmental competence, lower pregnancy success, and increased miscarriage [29, 30]. At the subcellular level, elevated Δ*Ψ*_m_ correlates with superior fertilization and blastocyst formation, whereas declines accompany oocyte aging [23, 31, 32]. GV chromatin configuration also predicts developmental potential, though human validation remains limited [33]. Overall, traditional morphological assessment retains immediate clinical relevance, but integration of functional and subcellular markers may enable more mechanism‐based and standardized embryo selection strategies in ARTs.

Importantly, the surrounding GCs, as key components of the follicular microenvironment, play a pivotal role in supporting oocyte maturation and endocrine function. During ovarian aging, GCs undergo multifaceted functional decline: their mitochondrial integrity deteriorates (reduced mitochondrial copy number, decreased membrane potential, elevated ROS, and mtDNA damage) which impairs metabolic homeostasis and compromises the energy and regulatory support provided to oocytes [20, 21]. Notably, the mevalonate (MVA) metabolic pathway in GCs is critically involved in regulating oocyte meiotic maturation. Robust MVA pathway activation in young GCs is suppressed in aged GCs, leading to meiotic defects and increased rates of aneuploidy. Supplementation with MVA intermediates (e.g., geranylgeraniol) rescues aged oocyte meiotic progression and improves euploidy and developmental competence [34]. Moreover, upregulation of malic enzyme 1 in senescent GCs suppresses the AMP‐activated protein kinase (AMPK)–FOXO1–IDH2 axis, triggering OS and activating the p53–p21 pathway, which accelerates GC senescence [35]. Conversely, exposing aged oocytes to young GCs restores mitochondrial activity and gene expression, improving development [36]. This highlights the crucial role of GCs in maintaining oocyte quality and suggests potential cell‐based therapies to combat age‐related decline.

In summary, age‐related alterations in oocyte biology arise from intricate cellular and microenvironmental mechanisms. Recent advances in functional assessment and GC‐based interventions offer promising avenues for enhancing outcomes in ARTs. These developments underscore the importance of adopting integrated approaches that incorporate both quantitative and qualitative dimensions in future fertility research.

## 3 Mechanisms of Ovarian Aging

**FIGURE 2:**
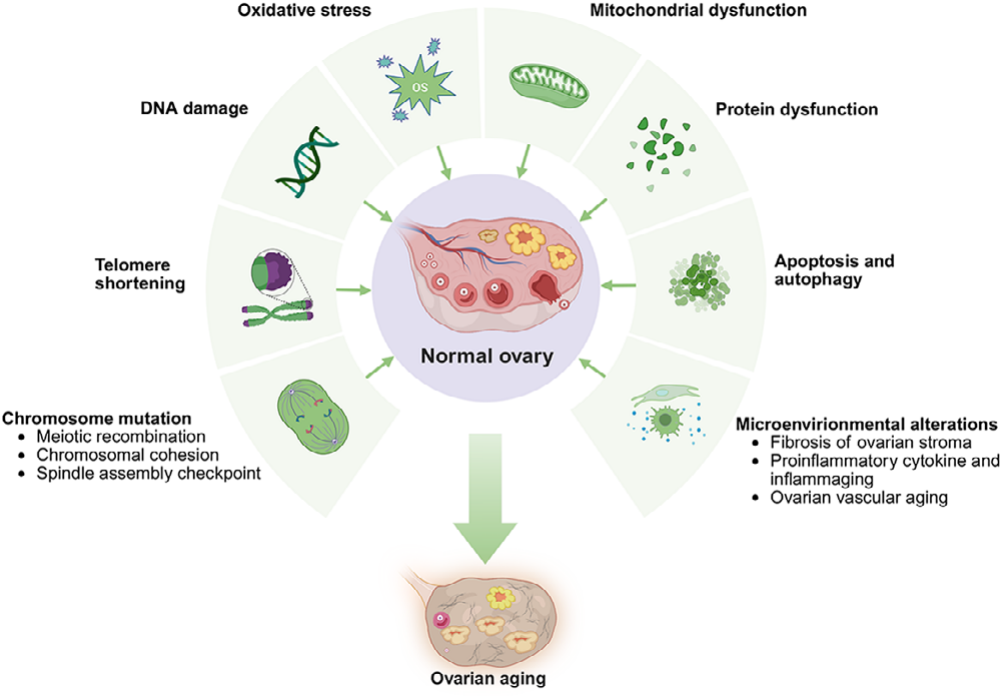
The mechanisms of the ovarian aging. Schematic overview of the major biological mechanisms driving ovarian aging, including genomic instability, metabolic and oxidative stress, and tissue microenvironment. Created with BioRender.com.

Ovarian aging results from intertwined molecular, metabolic, and environmental factors that progressively impair genomic integrity, cellular metabolism, and tissue homeostasis. Key mechanisms including chromosomal instability, telomere attrition, OS, mitochondrial dysfunction, and impaired proteostasis drive the decline in oocyte quality and accelerate follicular depletion. Concurrent alterations in the ovarian microenvironment, such as fibrosis, inflammation, and reduced vascularization, further compromise reproductive capacity. Lifestyle and environmental exposures also contribute to this multifactorial process, which extends beyond the ovary to systemic health. A comprehensive understanding of these mechanisms is critical for developing targeted therapies. The subsequent section systematically reviews these key drivers of ovarian aging (Figure 2).

### 3.1 Genomic Instability

#### 3.1.1 Chromosome Mutation

##### 3.1.1.1 Meiotic Recombination

The generation of haploid gametes requires a single round of DNA replication followed by two successive chromosome segregation events in a specialized form of cell division known as meiosis. A key step in meiosis is recombination, a process that involves the formation of DNA double‐strand breaks (DSBs) and their subsequent repair via crossover events, which are essential for ensuring genetic diversity and the structural integrity of the oocyte genome [37, 38]. Typically, each chromosome undergoes at least one crossover event [39]. However, the number and positioning of recombination events influence chromosome segregation fidelity and may contribute to aneuploidy [40]. A reduced frequency of recombination has been associated with maternal trisomy, with aberrations detected across multiple chromosomes [40]. Furthermore, recombination events occurring closer to the centromere become more frequent with advancing maternal age, increasing the likelihood of segregation errors [41]. By the end of a woman's reproductive lifespan, an estimated 50% of oocytes exhibit aneuploidy due to errors in recombination [42].

##### 3.1.1.2 Chromosomal Cohesion

The cohesion complex plays a critical role in meiosis, linking sister chromatids during DNA replication and ensuring accurate segregation of homologous chromosomes in meiosis I and sister chromatids in meiosis II [1, 43]. Studies in mice indicate that cohesion is established in the fetal ovary and remains unchanged until ovulation, suggesting that female fertility is directly linked to the lifespan of these cohesion proteins [44]. The meiosis‐specific cohesion subunits *SMC1β* and *REC8* are essential for chromosome segregation [45, 46]. Their expression is significantly reduced in oocytes from women aged 40 years compared with those aged 20 years [47]. However, transcriptome analyses reveal no significant difference in *Smc1β* expression between younger and older oocytes, suggesting posttranscriptional regulation [48]. Shugoshin 2 (SGO2), a key protector of centromeric cohesion, prevents premature cleavage by separase [49]. Age‐related loss of SGO2 has been observed, leading to weakened centromere cohesion and an increased incidence of aneuploidy [50]. Collectively, these findings suggest that age‐associated cohesion losses contribute to chromosomal missegregation, thereby reducing maternal fertility.

##### 3.1.1.3 Spindle Assembly Checkpoint

The spindle assembly checkpoint (SAC) safeguards genome integrity by regulating the timing and progression of cell division [41]. Monopolar spindle 1 (MPS1) kinase is a core component of the SAC [51]. Inhibition of SAC or MPS1 in young mouse oocytes recapitulates the phenotypic characteristics of aged oocytes, highlighting its role in age‐related chromosomal instability [51]. Other key SAC proteins, including budding‐uninhibited‐by‐benzimidazole 1 (BUB1) and its associated kinase BUBR1, exhibit reduced localization in aging oocytes, compromising checkpoint function and increasing susceptibility to aneuploidy [52]. However, while SAC dysfunction has been implicated in age‐related oocyte aneuploidy, evidence suggests that it plays a secondary role compared with defects in recombination and cohesion [16, 40].

#### 3.1.2 Telomere Shortening

Telomeres, composed of repetitive noncoding DNA sequences and associated binding proteins, cap the ends of eukaryotic chromosomes, safeguarding genomic integrity and chromosomal stability [53, 54]. Telomere length is closely related to reproductive lifespan and life expectancy [55]. A study of leukocyte telomere length found that each 1‐kilobase (kb) increase in telomere length corresponded to a 10.2‐month delay in the onset of natural menopause [56]. Women with the longest telomeres experienced menopause approximately 3 years later than those with the shortest telomeres, suggesting that leukocyte telomere length may serve as a predictive biomarker for menopausal timing [56]. In addition, telomere length may be associated with human aneuploid development [57]. Telomere DNA testing of polar bodies obtained by in vitro fertilization (IVF) revealed that aneuploid polar bodies had lower quantities of telomere DNA compared with that in paired sibling euploid polar bodies [57]. Another IVF‐based study suggested that telomere shortening may contribute to embryo fragmentation and preimplantation apoptosis, further linking telomere integrity to reproductive potential [58].

The progressive shortening of telomeres is largely attributed to diminished telomerase activity. Telomerase, a ribonucleoprotein enzyme complex composed of telomerase reverse transcriptase (TERT) and telomerase RNA component, counteracts telomere erosion by adding hexametric repeats to chromosomal termini [59]. Telomere‐associated proteins, including telomeric repeat binding factors 1 (TRF1) and 2 (TRF2) and protection of telomeres 1a (POT1a), safeguard telomere structure and function [60]. In human ovarian GCs and oocytes, the expression of TERT, TRF1, TRF2, and POT1 progressively declines from fetal development to menopause, coinciding with a reduction in telomere signal intensity [61]. Studies in telomerase‐knockout mice demonstrate progressive telomere attrition across successive generations, accelerating both systemic and reproductive aging [62]. In humans, GCs from patients with biochemical premature ovarian insufficiency (POI) exhibit lower telomerase activity compared with healthy controls [63], with similar reductions observed in patients with both occult and overt POI [64, 65]. As occult POI is often asymptomatic apart from subfertility, its clinical diagnosis remains challenging. Telomere length and telomerase activity may serve as valuable early biomarkers for predicting POI risk. Conversely, estrogen has been shown to regulate telomerase activity. Estrogen‐deficient mice exhibit suppressed telomerase function, resulting in accelerated telomere attrition and exacerbated ovarian aging [66]. By and large, the studies indicate that telomere shortening and reduced telomerase activity may significantly contribute to ovarian aging, with estrogen deficiency also adversely affecting telomere length. Further research is needed to explore the relationship between telomere dynamics and ovarian aging.

#### 3.1.3 DNA Damage

The DNA damage response (DDR) plays a pivotal role in preserving genomic integrity during reproductive aging, activating repair pathways to mitigate DNA lesions [67]. With advancing age, DDR efficiency declines, leading to the accumulation of DNA damage, increased oocyte attrition, and a heightened risk of infertility and miscarriage [16].

*BRCA1* and *BRCA2* are key genes involved in homologous recombination repair. Mutations in these genes increase the risk of breast and ovarian cancer. They are also linked to an earlier onset of menopause [1, 68]. Studies in rhesus monkeys indicate that DSB accumulation in follicles escalates with age, while *BRCA1* expression declines in GCs and oocytes, compromising DNA repair capacity [68]. Similarly, human oocytes from older individuals exhibit reduced *BRCA1* expression compared with those from younger counterparts [69]. *BRCA1* and *BRCA2* function within the DDR by facilitating DSB repair via the ataxia telangiectasia mutated (ATM) pathway [70]. Knockdown of ATM in oocytes results in an increased burden of unrepaired DSBs, decreased oocyte survival, and premature apoptosis [71]. Furthermore, women carrying *BRCA1* and *BRCA2* loss‐of‐function mutations experience menopause approximately 2.63 and 1.54 years earlier, respectively, than noncarriers [67].

The checkpoint kinase 2 (*CHEK2*) gene, another critical DDR component, is instrumental in maintaining genomic stability. A loss‐of‐function variant in *CHEK2* has been shown to delay natural menopause by 3.49 years and is associated with enhanced ovarian reserve [67, 72]. *CHEK2* phosphorylates and activates tumor protein p53 (*TP53*), orchestrating DDR outcomes [73]. Additionally, TAp63, a *TP53* isoform uniquely expressed in oocytes, triggers apoptosis in response to DNA damage upon activation by *CHEK2* [74]. These findings underscore the essential roles of *BRCA* and *CHEK2* in ovarian aging, highlighting their contributions to follicular depletion and variations in menopausal timing.

### 3.2 Metabolic and OS

#### 3.2.1 OS Caused by ROS and AGEs

ROS, primarily generated by mitochondria, serve as both signaling molecules and markers of ovarian aging, with their levels increasing as ovarian aging progresses [67, 75, 76]. OS arises when ROS production exceeds the capacity of cellular antioxidant defenses, leading to molecular damage [77]. Under normal conditions, cells eliminate excess ROS; however, with advancing age, antioxidant capacity declines [78]. This imbalance contributes to mitochondrial dysfunction, telomere attrition, and structural deterioration of oocytes and surrounding somatic cells, ultimately compromising ovarian function [79]. Furthermore, aged oocytes exhibit a diminished ability to generate antioxidants, exacerbating their vulnerability to OS‐induced damage [16]. Notably, reduced expression of the antioxidant enzyme superoxide dismutase 1 in aged mouse oocytes has been linked to increased OS and ferroptotic cell death in primordial follicles [78]. In GCs, elevated OS is associated with reduced follicle‐stimulating hormone receptor (FSHR) expression and impaired FSHR‐mediated signaling, potentially explaining the diminished ovarian response to elevated follicle‐stimulating hormone (FSH) in aging women [80]. The mechanisms underlying ROS dysregulation in ovarian aging remain incompletely understood. Emerging evidence suggests that abnormal iron metabolism may be a contributing factor [81]. In aged mice, oocyte iron accumulation correlates with increased oxidative damage, while iron chelation improves ovarian redox balance and enhances oocyte quantity and quality [81].

In addition to ROS, advanced glycation end products (AGEs) are another key driver of OS in the ovary. AGEs, formed through nonenzymatic glycation reactions, accumulate endogenously over time and can also be obtained from dietary sources [82]. Their accumulation in follicular fluid and serum has been implicated in impaired folliculogenesis, fertilization, and embryonic development [83]. AGEs may compromise oocyte quality by triggering inflammatory responses within the follicular microenvironment [84]. Upon binding to AGEs receptors, AGEs activate nuclear factor kappa B (NF‐κB) and nicotinamide adenine dinucleotide phosphate (NADPH) oxidase, promoting ROS generation [85]. In turn, OS accelerates the final steps of advanced glycation, creating a deleterious feedback loop [86]. Given the intricate interplay between AGEs and ROS, their cumulative effects likely play a pivotal role in OS‐induced ovarian aging.

#### 3.2.2 Mitochondrial Dysfunction

Mitochondria, essential for ATP production and cellular metabolism, undergo age‐related structural and functional deterioration in oocytes. In older women, mitochondrial density and number decline, while their morphology becomes increasingly aberrant [87]. During oocyte maturation, mtDNA copy number increases dramatically, supporting the energy demands of meiotic progression [88]. However, age‐related ROS accumulation directly damages mtDNA, leading to DSBs and impairments in ATP synthesis. To repair these breaks, poly ADP‐ribose polymerase 1 (PARP1) is activated, depleting cellular pools of nicotinamide adenine dinucleotide (NAD⁺) and ATP, thereby perpetuating a cycle of mitochondrial dysfunction [21]. Mitochondria continuously divide and replicate throughout life, but with age, replication errors accumulate, leading to a progressive increase in mtDNA mutations [21, 89]. Compounding this issue, the efficiency of mtDNA repair mechanisms declines over time, accelerating mitochondrial genomic instability. The interdependence of ROS generation and mtDNA damage exacerbates mitochondrial dysfunction, ultimately compromising both ovarian function and systemic health. Single‐cell transcriptomic analysis of ovarian cells from young and aged nonhuman primates highlights oxidative damage as a critical factor in the age‐related decline of ovarian function [90].

#### 3.2.3 Apoptosis and Autophagy

Apoptosis and autophagy are two forms of programmed cell death, that being fundamental to tissue homeostasis and organismal development [18]. Apoptosis occurs via two primary pathways: the intrinsic (mitochondrial) pathway, regulated by intracellular signals, and the extrinsic pathway, triggered by extracellular factors [91]. GCls play a pivotal role in oocyte survival by supplying essential nutrients and growth factors while also shielding oocytes from OS [92]. GC apoptosis is a key driver of follicular atresia [93]. Gene expression analysis in nonhuman primate GCs revealed that age‐related upregulation of 62 genes was predominantly associated with positive regulation of apoptotic pathways [90]. Apoptosis is also associated with a decrease in follicle number. It has been shown that deficiency of the proapoptotic factor BAX maintains primordial follicle numbers to some extent and prolongs fertility [18]. However, a study found that the overexpression of the antiapoptotic protein BXL‐2 increased follicle number in newborn mice but had no effect on the ovaries of adult mice [94]. It remains to be seen whether this positive effect in newborn mice extends ovarian lifespan.

Like apoptosis, autophagy is a highly regulated process of cellular degradation, characterized by the sequestration of cytoplasmic components into autophagic vacuoles for lysosomal degradation [92]. Under physiological conditions, autophagy maintains cellular homeostasis by removing damaged organelles and proteins. Ovarian aging may be related to excessive autophagy that can promote apoptosis and accelerate follicular depletion [95]. During in vitro maturation of porcine oocytes, aberrant autophagic activation disrupted mitochondrial and autophagosome distribution, leading to compromised oocyte viability, suggesting a role for autophagy in oocyte quality control [96]. However, other studies indicate that ovarian aging is associated with decreased autophagy. Reduced expression of genes associated with autophagy initiation and maintenance was found in aged rat models [97]. Another study found that the expression of nerve growth factor inducible gene B (Nur77) was reduced in the ovaries of aging mice. Overexpression of Nur77 activates mitochondrial autophagy, reduces apoptosis, and regulates follicular number and hormonal abnormalities caused by ovarian aging, ultimately improving ovarian function [98]. These apparently conflicting findings may reflect differences in species and experimental models (e.g., porcine in vitro systems versus rodent in vivo studies), the stage‐specific roles of autophagy (protective at basal levels but detrimental when hyperactivated), and methodological variation in measuring autophagic flux (gene expression versus functional assays). A synthesized perspective suggests that basal autophagy is vital for oocyte quality, whereas both insufficient and excessive activation can impair ovarian function depending on context, calling for more research to delineate thresholds and regulatory mechanisms across models.

In summary, the paradoxical role of autophagy in ovarian aging can be reconciled through the principle of homeostatic control. As a fundamental quality‐control mechanism, autophagy is neither inherently protective nor detrimental; its functional outcome depends on the precision of its regulation. An age‐related decline promotes the accumulation of cellular damage, while excessive activation can trigger cell death. Consequently, ovarian aging may stem primarily from a loss of autophagic balance rather than a simple deficit or surplus. Future therapeutic strategies should therefore aim to restore this homeostatic set‐point, not nonspecifically enhance or inhibit autophagy.

#### 3.2.4 Protein Metabolism Dysregulation

Protein homeostasis is essential for cellular function and is maintained through a finely tuned network of synthesis, folding, modification, and degradation. However, aging disrupts this balance, leading to the accumulation of misfolded or damaged proteins [92]. In aged mouse oocytes, dysregulated protein metabolism impairs oocyte quality and accelerates reproductive senescence. Comparative transcriptomic analyses have shown that genes involved in protein modification and metabolism are significantly enriched in young follicles, suggesting that younger oocytes possess a more robust protein quality control system [99]. Proper protein folding is a crucial step in proteostasis. Misfolded proteins activate the unfolded protein response (UPR) in both the endoplasmic reticulum and mitochondria [92]. Failure to restore proteostasis triggers excessive endoplasmic reticulum stress, ultimately inducing apoptosis [100]. The mitochondrial caseinolytic peptidase P (CLPP) is a key enzyme that degrades misfolded proteins. Knockout of CLPP in mice resulted in accelerated depletion of the ovarian reserve and a significant reduction in oocyte number, highlighting the essential role of proteostasis in ovarian lifespan [101]. Mitochondrial ATP‐dependent Lon protease 1 (LONP1) is another critical protease that safeguards mitochondrial proteostasis by degrading misfolded proteins, regulating mtDNA replication, and stabilizing the electron transport chain [102, 103, 104]. Age‐dependent alterations in LONP1 expression have been observed in animal models, implicating it in ovarian aging [105]. Conditional ablation of LONP1 in mouse oocytes impairs follicular progression from the secondary to antral stage, triggers persistent activation of the mitochondrial UPR, and culminates in infertility [106]. These findings establish LONP1 as a gatekeeper of mitochondrial function and oocyte survival. Beyond aging, proteostasis regulators intersect with disease states. Notably, the interaction between LONP1 and cytochrome P450 family 11 subfamily A member 1 has been implicated in polycystic ovary syndrome (PCOS), and pharmacological interventions such as artemisinin have been shown to alleviate PCOS phenotypes by enhancing this interaction [107]. These insights position proteostasis not only as a determinant of ovarian aging but also as a potential therapeutic target for reproductive disorders.

### 3.3 Tissue Microenvironment

#### 3.3.1 Fibrosis of Ovarian Stroma

Fibrosis is not only a hallmark of tissue aging, but also a crucial factor leading to structural and functional deterioration. Among the canonical pathways implicated in fibrogenesis, the transforming growth factor β (TGF‐β)/Samd and IL‐6/Janus kinase (JAK)–signal transducer and activator of transcription (STAT) cascades occupy central roles. In the ovary, studies in PCOS patients have demonstrated that aberrant activation of the TGF‐β/Smad pathway promotes stromal fibrosis [108, 109]. Evidence further suggests that FSH/FSHR signaling enhances TGF‐β1/Smad activity, linking endocrine dysregulation to fibrotic remodeling. Inhibition of FSH, FSHR, or TGF‐β1 attenuates fibrosis and restores ovarian reserve [110]. Besides, the JAK–STAT signaling pathway is also observed in the fibrosis process of other organs such as the heart and ureter [111, 112]. However, the IL‐6/JAK–STAT axis remains underexplored in the context of ovarian aging. Picrosirius Red staining reveals a significant increase in stromal fibrosis in mice at 7 and 9 months of age compared with younger counterparts [113]. Moreover, the extent of fibrosis correlates with chronological age, becoming more pronounced with ovarian aging [113]. Within the extracellular matrix (ECM), age‐related collagen deposition contributes to increased ovarian stiffness [114]. In contrast, levels of hyaluronan (HA), a key ECM component, decline with age, potentially due to dysregulated expression of HA synthases and hyaluronidases [114]. This age‐related collagen deposition and HA reduction increases ovarian stiffness [114]. Follicle expansion requires a soft ECM. This age‐related ECM remodeling limits follicle expansion and disrupts mechanical signaling pathways (primarily mechanosensitive pathways such as Hippo and PI3K/AKT), which convert mechanical signals into biochemical responses ultimately linked to follicle fate [115, 116]. Additionally, ovarian hypoxia caused by defects in the construction of the ovarian vascular network due to age‐related ovarian fibrosis also affects follicle growth and development [117]. Studies have shown that ovarian fibrosis induced by premature ovarian failure (POF) is closely associated with a reduction in vascular distribution within ovarian stromal tissue [118]. The decrease in vascular density and collagen deposition may impede the diffusion of nutrients and oxygen. In summary, ovarian fibrosis disrupts the balance between follicular dormancy and activation, impairing ovarian function through multiple pathways. These alterations in stromal composition may impair follicular development and contribute to ovarian aging. The mechanisms by which fibrosis contributes to ovarian aging require further investigation.

#### 3.3.2 Proinflammatory Cytokine and Inflammaging

Aging is associated with chronic low‐grade inflammation, a phenomenon often referred to as “inflammaging,” which is linked to tissue fibrosis and functional decline [16]. The ovarian follicular microenvironment is no exception. In human follicular fluid, levels of specific proinflammatory cytokines—including IL‐3, IL‐7, IL‐15, TGF‐β1, and MIP‐1—increase with age and inversely correlate with AMH levels [119]. Notably, the age‐related elevation of TGF‐β3 appears to be unique to the ovarian stroma, implicating stromal inflammation as a key contributor to ovarian aging [119]. Inflammation has a profound impact on both oocyte quality and follicular depletion [113, 120]. Studies in knockout mouse models of proinflammatory cytokines have demonstrated that genetic ablation of specific inflammatory mediators extends reproductive lifespan and preserves ovarian reserve [121, 122].

Macrophages, the predominant immune cells in the ovary, play a dual role in ovarian physiology [123]. The balance between proinflammatory M1 and anti‐inflammatory M2 macrophages is critical for maintaining ovarian function [123]. With advancing age, expression of M1‐associated markers declines, whereas M2‐associated markers increase, indicating a shift in polarization from M1 toward M2 during ovarian aging [124]. Inhibition of M2 macrophage polarization in aging mice attenuated ovarian fibrosis and improved ovarian function and fertility [125]. This may be because anti‐inflammatory M2 macrophages stimulate fibroblasts to produce collagen, and appropriate collagen promotes tissue repair, while excessive collagen deposition promotes fibrosis [126]. In addition, compared with monocyte‐derived macrophages, the proportion of tissue‐resident macrophages was significantly reduced in aged ovaries, which seems to be the reason for the shift of macrophage polarization in ovaries [124, 127, 128]. Furthermore, suppression of proinflammatory signaling in aged ovaries reduced macrophage infiltration and restored ovarian function [129]. Chronic low‐grade inflammation from proinflammatory factors and macrophages may accelerate ovarian aging through fibrosis and dysfunction. Further research is needed to explore specific signaling pathways and target factors.

#### 3.3.3 Ovarian Vascular Aging

Ovarian vascular aging has recently emerged as a critical yet understudied contributor to ovarian decline. The maintenance of ovarian function in adult women depends on continuous vascular remodeling, which supports follicular growth and ovulation [130]. Temporarily inhibiting vascularization in the ovaries of young women using axitinib can reduce follicle depletion, maintain long‐term ovarian reserve, and ultimately delay ovarian aging [131]. Recent studies identify endothelial cell aging as a central driver of impaired ovarian vascularization. Age‐related reductions in vascular remodeling limit nutrient and oxygen delivery, thereby compromising follicle development. GC‐derived vascular endothelial growth factor A (VEGFA) is pivotal in sustaining ovarian angiogenesis. Declines in VEGFA expression with age correlate with diminished vascular formation and reduced ovarian perfusion. Transcriptomic comparisons between young and middle‐aged ovaries further implicate endothelial dysfunction in the deterioration of vascular support. Supplementing with salidroside can enhance ovarian vascular function and improve fertility in older women [132]. This provides new avenues for exploring vascular‐targeted therapies as a strategy to extend reproductive healthspan.

### 3.4 Others

Beyond intrinsic molecular mechanisms, ovarian aging is strongly shaped by external and environmental factors. Lifestyle and dietary habits, in particular, exert a profound influence on the pace of ovarian decline and the timing of natural menopause. Epidemiological studies suggest that increased consumption of green vegetables correlates with later menopause, while higher intake of plant protein and fruit is similarly associated with delayed ovarian aging [133, 134]. In contrast, more nuanced analyses reveal that adherence to a healthy plant‐based diet does not significantly shift menopausal timing, whereas unhealthy plant‐based patterns may accelerate reproductive senescence [135]. In humans, data indicate that consuming foods rich in plant estrogens, such as soy isoflavones, may suppress estrogen and progesterone concentrations and cause a surge in luteinizing hormone (LH) and FSH before ovulation, thereby delaying menstruation [136]. Additionally, high‐fat diets represent another area of concern. In animal models, oocytes from high‐fat‐fed mice accumulate excess lipids, while obese women show elevated triglyceride concentrations in follicular fluid, indicating a shared metabolic signature [137, 138]. A study of South African women showed that when dietary fat intake increased by 5% over 2 months, FSH levels rose, estrogen decreased, and the follicular phase lengthened [136].

Exercise and smoking highlight the duality of lifestyle effects. Women who engage in regular physical activity often experience menopause later than sedentary women [139]. However, rapid introduction of intense exercise may be associated with menstrual cycle disorders [140]. Direct evidence to for its role in POI remains limited [136]. Smoking, by contrast, is unequivocally detrimental. Toxins in cigarette smoke impair endocrine balance, damage oocyte quality, accelerate ovarian reserve depletion, and are strongly associated with early menopause and POI [141, 142].

Additional determinants, including psychological stress, ethnicity, excessive weight loss, and high‐carbohydrate diets, have also been implicated in modulating ovarian aging [143]. Endocrine‐disrupting chemicals, encountered through contaminated food, water, air, cosmetic use, or occupational exposure (e.g., pesticides), interfere with hormonal signaling and further compromise ovarian function [144]. Overall, these findings emphasize that ovarian aging is not solely a product of intrinsic cellular decline but rather a multifactorial process shaped by complex interactions between biology, lifestyle, and environment.

Collectively, these findings reveal ovarian aging as a dynamic process driven by genetic instability, metabolic stress, microenvironmental remodeling, and lifestyle determinants. Despite advances in elucidating key pathways, questions remain regarding their relative contributions and therapeutic translation. Future research integrating molecular, environmental, and clinical perspectives will be crucial to developing effective strategies to preserve fertility, delay menopause, and reduce associated health risks.

## 4 Association of Ovarian Aging With Age‐Related Diseases

**FIGURE 3:**
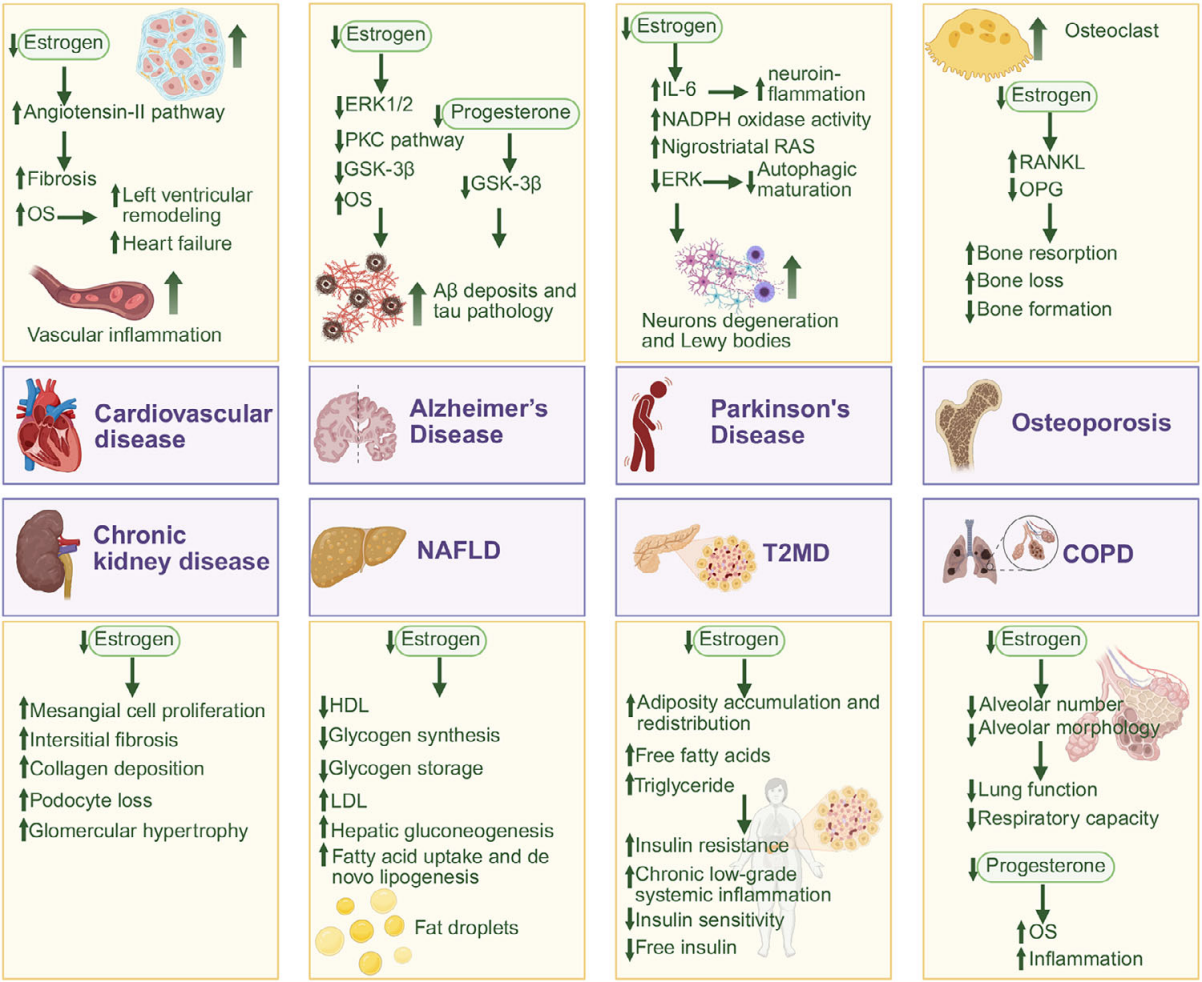
The association between ovarian aging and age‐related disease. Diagram summarizing the links between ovarian aging and systemic disorders, such as cardiovascular disease, osteoporosis, neurodegeneration, and metabolic dysfunction. Created with BioRender.com.

As ligand‐activated transcription factors, ERs exhibit ubiquitous expression across multiple physiological systems, mediating the pleiotropic effects of ovarian‐derived estrogen in maintaining tissue homeostasis and regulating organ functionality. The age‐related decline in ovarian reserve leads to systemic estrogen deficiency, thereby forming a pathophysiological foundation for a range of age‐associated disorders. The age‐related decline in ovarian reserve leads to systemic estrogen deficiency, thereby forming a pathophysiological foundation for a range of age‐associated disorders. This section will systematically elucidate the mechanistic correlations between ovarian aging and the pathogenesis of cardiometabolic, neurodegenerative, osteoarticular, and other age‐related diseases, with a focused emphasis on estrogen‐dependent regulatory pathways (Figure 3). Based on evidence strength and disease burden in postmenopausal women, these conditions are categorized into core and potential age‐related diseases, reflecting their differential epidemiological and clinical relevance (Table S2).

### 4.1 Core Age‐Related Diseases

#### 4.1.1 Ovarian Aging on CVD

Estrogen exerts multiple cardioprotective effects, which decline with ovarian aging. In aging ovariectomized rats, estrogen has been shown to attenuate left ventricular remodeling, potentially by suppressing fibrotic responses and inhibiting myofibroblast differentiation via downregulation of the angiotensin‐II pathway [145, 146]. In postmenopausal women, estrogen deficiency exacerbates OS, contributing to left ventricular hypertrophy, which in turn increases the risk of diastolic dysfunction and heart failure with preserved ejection fraction [147]. Furthermore, reduced estrogen levels are associated with a decline in cardiomyocyte sarcoplasmic ATP‐sensitive K⁺ (K_ATP_) channels, decreasing myocardial stress tolerance in older women [148]. Beyond its direct myocardial effects, estrogen modulates vascular homeostasis by regulating chemokine and cytokine expression following vascular injury and limiting leukocyte infiltration [149]. It downregulates neutrophil chemokines, reduces C‐reactive protein levels in damaged arteries, and suppresses ROS production, thereby mitigating vascular inflammation and oxidative damage [150, 151]. However, the loss of estrogen after menopause leads to a decline in these protective mechanisms, contributing to a significant rise in cardiovascular morbidity [152].

The link between ovarian aging and CVD has been substantiated by multiple clinical studies (Table 1). The European Prospective Investigation into Cancer and Nutrition‐CVD study, which included 15,402 European women aged 35–70 years at baseline, demonstrated a linear association between earlier menopause and increased coronary heart disease (CHD) risk, with an adjusted hazard ratio (HR) of 1.02 per year decrease in menopausal age (*p* = 0.001) [153]. Additionally, women who underwent surgical menopause experienced a higher risk of CHD compared with those with natural menopause (adjusted HR 1.25[95% confidence interval [CI], 1.10–1.42], *p* < 0.001) [153]. A 2016 pooled meta‐analysis of 32 observational studies involving 310,329 nonoverlapping women observed similar findings. The risks of overall (relative risk [RR], 1.50 [95% CI, 1.28–1.76]) and fatal CHD (RR, 1.11 [1.03–1.20]) escalated in women with early menopause (<45 years) compared with women with menopause at the age of 45 years or older [154]. Moreover, women with early menopause (<45 years) encountered a significantly greater risk of heart failure than those with later menopause, as shown by a meta‐analysis of three prospective studies involving 3,568 heart failure cases (HR, 1.33 [1.15–1.53]) [155]. A cross‐sectional study enrolled 2037 Korean women aged 44–56 years across various menopausal stages (premenopausal, early menopause transition [MT], late MT, and postmenopausal) found significantly higher blood pressure in late MT compared with early MT [156]. Longitudinal assessments of carotid artery morphology during the menopausal transition further demonstrated that carotid intima‐media thickness and adventitia diameter increased most prominently in the late perimenopausal stage, relative to premenopausal and early perimenopausal stages [157]. Over a mean follow‐up of 2.3 years, aortic pulse wave velocity was observed to increase more rapidly in middle‐aged women undergoing menopause than in premenopausal or postmenopausal women [158]. Overall, these findings underscore the critical impact of ovarian aging on cardiovascular health, with the menopausal transition representing a period of heightened CVD risk.

**TABLE 1:** The impacts of ovarian aging on multiple organ age‐related diseases.

Age‐related diseases	Specific manifestations	Epidemiological evidence	References
Cardiovascular disease	Accelerate left ventricular remodeling and hypertrophy [145, 147] Decrease myocardial tress tolerance [148] Aggravate vascular inflammation and oxidative damage [150, 151]	Earlier menopause increased the risk of coronary heart disease (*p* = 0.001).	[153]
		Women underwent surgical menopause experienced a higher risk of coronary heart disease (adjusted HR, 1.25 [95% CI, 1.10–1.42], *p* < 0.001).	[153]
		The risk of overall (RR, 1.50 [95% CI, 1.28–1.76]) and fatal CHD (RR, 1.11 [95% CI, 1.03–1.20]) escalated in women with early menopause (<45 years).	[154]
		Women with early menopause (<45 years) encountered a greater risk of heart failure than those with later menopause (HR, 1.33 [95% CI, 1.15‐1.53]).	[155]
		The blood pressure was higher in late MT compared with early MT.	[156]
		Carotid intima‐media thickness and adventitia diameter increased most prominently in the late perimenopausal stage.	[157]
		Aortic pulse wave velocity increased more rapidly in women undergoing menopause than in premenopausal or postmenopausal women.	[158]
Alzheimer's disease	Increase the formation of Aβ and tau pathology [159, 160] Disrupts bioenergetics, inflammation, and lipid profiles in various brain systems [161, 162, 163] Impair cognitive ability [164]	Women experiencing early menopause had higher odds of verbal fluency, visual memory, and psychomotor speed decline (ORs, 1.51, 1.43, and 1.36, respectively).	[165]
		Prolonged childbearing and delayed menopause were associated with a decreased risk of AD (OR, 0.96 and 0.949, respectively).	[166]
		Bilateral oophorectomy performed before natural menopause elevates the risk of MCI (adjusted OR, 2.21, *p* < 0.001).	[167]
Parkinson's disease	Promote neuroinflammation and neuronal degeneration [168, 169, 170]	Age at menopause had inverse association with the risk of PD (OR, 0.84, [95% CI, 0.73–0.98], *p* = 0.03).	[171]
		Age at menopause and age at PD onset had positive correlation (*r* = 0.55, *p *= 0.001).	[172]
		Later menopause and delayed PD onset had positive correlation (*p* < 0.01).	[173]
		Female with early menopause had a higher rate on PD than age‐matched female controls (24 vs. 16%).	[174]
		Bilateral oophorectomy was significantly associated with the increased risk of PD (OR, 3.55, [95% CI, 1.75–7.20]).	[175]
Osteoporosis	Disrupt bone homeostasis [176] Contribute to progressive bone loss [177]	Osteoporosis prevalence is 19.6 and 27.1% in women over 50 years and over 65 years, 4.4 and 5.7% in age‐matched men.	[178]
		Cortical bone and trabecular bone loss most rapidly around menopause.	[179]
		71% of osteoporotic fractures bone loss begins 1–3 years before menopause at an annual rate of about 2%, persisting for 5–10 years postmenopause.	[180, 181]
Chronic kidney disease	Aggravate glomerular structural malformations [182, 183] Impact sodium and water balance [184]	Women experiencing early menopause before age 45 had an increased risk of CKD compared with women experiencing natural menopause at age ≥45 years (OR = 1.26, [95% CI, 1.01–1.56]).	[185]
		Women with bilateral oophorectomy had a higher risk of CKD (HR = 1.42, [95% CI, 1.14–1.77]).	[6]
Nonalcoholic fatty liver disease	Promote hepatic gluconeogenesis [186] Accelerate hepatic lipid accumulation [187] Exacerbate oxidative stress and hepatic inflammation [188]	60% prevalence of NAFLD in postmenopausal women compared with 32% in premenopausal women.	[189]
		NAFLD prevalence rises from 42.9% in premenopausal obese women to 60.2% in postmenopausal counterparts.	[190]
Type 2 diabetes mellitus	Promote peripheral insulin resistance and chronic low‐grade systemic inflammation [191] Reduce insulin sensitivity [191] Contribute to progressive β‐cell failure [192]	Women with POF faced a 32% higher risk of T2DM compared with those with normal ovarian function.	[193]
		A shorter reproductive lifespan correlated with a higher T2DM risk (HR = 1.06, 95% CI = 1.01–1.12).	[193]
		Women with a reproductive lifespan less than 30 years exhibited a 37% greater risk of T2DM compared with women with a reproductive span of 36–40 years.	[194]
		Each year of reproductive lifespan reduction was associated with a 6–15% increase in T2DM risk.	[195]
Chronic obstructive pulmonary disease	Reduce alveolar number [196] Destruct alveolar architecture [197] Increase oxidative damage and inflammation in bronchoalveolar [198]	Postmenopausal women experienced reduced FVC, FEV_1_, and 25–75% forced expiratory flow (FEF_25–75_).	[199]
		Menopausal women had diminished FVC and FEV_1_, along with an increased risk of pulmonary impairment (FVC < lower limit of normal).	[200]
		Those who experienced menopause before the age of 47 years had an elevated risk of COPD‐related hospitalization or mortality.	[201]
		Menopause age and COPD risk had an inverse relationship.	[202]

#### 4.1.2 Ovarian Aging on Alzheimer's Disease

Alzheimer's disease (AD) is an age‐related neurodegenerative disorder characterized by progressive dementia, functional impairment, and a diminished quality of life. The hallmark pathological features of AD include extracellular amyloid‐β (Aβ) deposition and intracellular tau neurofibrillary tangles (NFTs) [159, 160]. New evidence indicates that ovarian aging and the resulting decrease in sex steroid hormones may influence AD pathogenesis by affecting Aβ and tau pathology, potentially enhancing their toxicity. In female triple transgenic AD (3xTgAD) mice, ovariectomy‐induced hormone depletion led to a substantial increase in Aβ accumulation and cognitive impairment [203]. Treatment with estradiol induced a decrease in Aβ levels and aggregation into plaques in female mice expressing the Swedish and Indiana mutations of human amyloid precursor protein (APP) compared with wild‐type mice [204]. Estrogen may influence Aβ levels by shifting APP processing toward the nonamyloidogenic (α‐secretase) pathway via the activation of extracellularly regulated kinases 1 and 2 (ERK1/2) and protein kinase C signaling [203]. Additionally, ERK1/2 activation inhibits γ‐secretase, thereby downregulating β‐site APP‐cleaving enzyme 1 (BACE1) and reducing Aβ production [203]. Given that OS enhances BACE1 activity, estradiol may further suppress Aβ accumulation by stimulating antioxidant defenses and reinforcing ERK1/2‐mediated inhibition of γ‐secretase and BACE1 [203]. NFTs, composed of hyperphosphorylated tau aggregates, represent another defining histopathological feature of AD. In vitro studies indicate that estradiol decreases protein kinase A activity, an enzyme involved in tau phosphorylation, and rescues abnormal tau proteins [203]. In 3xTgAD female mice, both continuous and cyclic administration of estradiol and progesterone mitigated tau hyperphosphorylation. This protective effect may stem from estradiol‐mediated inhibition of glycogen synthase kinase 3β (GSK‐3β), a key enzyme in tau hyperphosphorylation, and progesterone‐induced suppression of GSK‐3β expression and tau protein levels [203]. Taken together, these findings demonstrated that sex hormones, especially estrogen, are able to modulate AD‐related neuropathology and preserve cognitive function.

Mounting evidence coming from human studies also elucidated the impacts of ovarian aging on AD. The prevalence of AD is twice as high in women as in men at the age of 65 years [205]. The menopausal transition may contribute to this increased susceptibility in women, as the dysregulation of neuromodulatory hormones, such as estrogen, disrupts various brain systems, including bioenergetics, inflammation, and lipid profiles [161, 162, 163]. Changes in hormone profiles directly impact brain function and can be used as predictors of cognitive decline, with the estrogen to FSH ratio specifically indicating mild cognitive impairment (MCI) [164]. Furthermore, earlier menopause has been associated with an increased risk of AD and accelerated cognitive decline. One study reported that women experiencing early menopause had higher odds of verbal fluency, visual memory, and psychomotor speed decline (odds ratios [ORs] of 1.51, 1.43, and 1.36, respectively), with an overall HR of 1.35 for AD [165]. Xi et al. [166] observed similar findings. They noted that prolonged childbearing and delayed menopause were associated with a decreased risk of AD (OR = 0.96 and 0.949, respectively) [166]. Moreover, bilateral oophorectomy performed before natural menopause significantly elevates the risk of MCI, with an adjusted OR of 2.21 (*p* < 0.001) [167]. Surgical menopause at an earlier age correlated with a more rapid decline in cognitive function, particularly in domains such as episodic and semantic memory [206]. Ovarian aging and the associated decrease in estrogen may contribute to Aβ accumulation and tau protein hyperphosphorylation, resulting in cognitive decline. More high‐quality clinical data are necessary to strengthen the evidence linking ovarian aging to AD.

#### 4.1.3 Ovarian Aging on Parkinson's Disease

Parkinson's disease (PD), the second most common neurodegenerative disorder, is marked by the progressive degeneration of dopamine‐producing neurons in the substantia nigra and the formation of Lewy bodies, which are intraneuronal protein aggregates primarily composed of α‐synuclein [207]. Accumulating evidence suggests that estrogen exerts neuroprotective effects on the nigrostriatal dopaminergic system, mitigating neuronal degeneration through multiple mechanisms. These include modulation of monoamine oxidase activity, inhibition of IL‐6 to suppress neuroinflammation, activation of ERK signaling to enhance autophagic maturation in dopaminergic brain regions, and downregulation of both the nigrostriatal renin–angiotensin system and NADPH oxidase activity [168, 169, 170]. In nonhuman primates, prolonged estrogen deprivation leads to irreversible dopaminergic neurodegeneration. Within 30 days of ovariectomy, over 30% of nigrostriatal dopaminergic neurons are permanently lost [208]. Notably, transient estrogen replacement restores tyrosine hydroxylase‐immunoreactive cell densities when initiated within 10 days of ovariectomy but fails to do so after 30 days, underscoring the critical temporal window for estrogen‐mediated neuroprotection [208].

Data from human studies observed similar findings on estrogen deprivation. Using a Mendelian randomization approach, Kusters et al. [171] identified an inverse association between age at menopause and risk of PD, reporting an OR of 0.84 (95% CI, 0.73–0.98; *p* = 0.03) per year increase in menopausal age. Other studies have confirmed this linear association. A cross‐sectional study recruited women with PD and age matched healthy women controls indicated a significant positive correlation between age at menopause and age at PD onset (*r* = 0.55, *p *= 0.001) [172]. Similarly, Frentzel et al. [173] analyzed age‐matched male and female PD patients, demonstrating a positive correlation between later menopause and delayed PD onset (*p* < 0.01). Moreover, a higher rate of early menopause was observed in female PD cohorts than in age‐matched female controls (24 vs. 16%) [174]. Surgical menopause further exacerbates PD risk. A recent study by Canonico et al. [175] revealed that bilateral oophorectomy, but not hysterectomy, was significantly associated with the increased risk of PD (OR = 3.55, 95% CI = 1.75–7.20). In summary, both earlier natural menopause and surgical menopause heighten the risk of PD, likely due to the abrupt or premature decline in estrogen levels.

#### 4.1.4 Ovarian Aging on Osteoporosis

Osteoporosis is the most prevalent age‐related bone disease, characterized by low bone mass and disrupted bone microarchitecture, resulting in increased fragility and fracture risk [178]. This condition arises from an imbalance between osteoblast‐mediated bone formation and osteoclast‐mediated bone resorption, disrupting skeletal homeostasis [176]. In postmenopausal osteoporosis, estrogen deficiency accelerates bone resorption while impairing bone formation, leading to progressive bone loss [177]. A key mechanism underlying estrogen‐mediated bone homeostasis involves the receptor activator of NF‐κB (RANK)–RANK ligand (RANKL)–osteoprotegerin (OPG) signaling pathway [209]. Estrogen deficiency dysregulates this pathway by increasing RANKL expression in stromal cells and lymphocytes, thereby enhancing osteoclast sensitivity to RANKL and promoting osteoclast differentiation and activation [177]. Concurrently, diminished estrogen levels reduce OPG secretion from osteoblasts, weakening OPG's inhibitory effect on RANKL‐induced osteoclast activation [177]. As a result, ovarian aging drives excessive bone resorption, culminating in net bone loss and skeletal fragility.

The detrimental effects of ovarian aging on bone metabolism are particularly evident during the MT, as confirmed by epidemiological data. Osteoporosis prevalence in women surpasses that in men, affecting 19.6% of women over 50 years and 27.1% of those over 65 years, compared with 4.4 and 5.7%, respectively, in age‐matched men [178]. Notably, women account for 71% of osteoporotic fractures [180]. Bone mass peaks around the age of 30 years, after which women lose approximately 30% of cortical bone and 50% of trabecular bone over their lifetime, with the most rapid loss occurring around menopause [179]. While premenopausal women maintain relatively stable bone mass, bone loss begins 1–3 years before menopause at an annual rate of about 2%, persisting for 5–10 years postmenopause [181]. Nearly half of lifetime bone loss occurs within the first 5 years following menopause, with the steepest decline beginning 2 years before the final menstrual period [210]. After this phase, bone density continues to decline at approximately 0.5% per year, resulting in an average 30% reduction in bone mass by the age of 80 years compared with its peak [181]. The clinical implications of postmenopausal osteoporosis are profound. Globally, one in three women over 50 years of age will experience an osteoporotic fracture [211]. Hip fractures, in particular, carry severe consequences, with a mortality rate exceeding that of breast, uterine, and ovarian cancers combined [212]. Hospitalization is often required, and up to 25% of hip fracture patients require long‐term care, while 50% suffer permanent mobility loss [181]. This highlights the significant threat of osteoporosis related to ovarian aging and the need for preventive and therapeutic measures.

### 4.2 Potentially Age‐Related Diseases

#### 4.2.1 Ovarian Aging on CKD

CKD is a progressive condition characterized by deteriorating kidney function, persistent structural abnormalities, and other indicators of kidney impairment [213]. Estrogen is essential for maintaining kidney normal structure and function, exerting protective effects through multiple mechanisms. It prevents glomerular structural malformations by inhibiting mesangial cell proliferation, reducing collagen deposition and interstitial fibrosis, and mitigating podocyte loss and glomerular hypertrophy [182, 183]. Additionally, estrogen suppresses glomerulosclerosis by reducing the synthesis of type I and type IV collagen and preventing excessive ECM accumulation [214]. Estrogen also regulates kidney homeostasis through its influence on sodium and water balance. It downregulates the expression and activity of α‐, β‐, and γ‐subunits of the epithelial sodium channel and α‐sodium/potassium adenosine triphosphatase (α‐Na/K‐ATPase), while increasing the expression of angiotensin type 2 receptor and inhibiting aldosterone secretion, which regulate osmoregulation and water–electrolyte homeostasis [184]. Estrogen modulates blood pressure by influencing the renin–angiotensin–aldosterone system and regulates erythrocyte levels either directly by affecting erythropoietin (EPO) production or indirectly by altering the tissue‐specific response to EPO [184]. Notably, estrogen therapy in ovariectomized mice has been shown to attenuate renal fibrosis associated with kidney dysfunction [215]. Taken together, estrogen deficiency resulting from ovarian aging may exacerbate structural and functional renal decline.

Human studies show a linear increase in CKD prevalence with age, rising from 13.7% in the 30–40 years age group to 34.3% in the 70–80 years age group [216]. Women have a higher risk of CKD than men, with 15.3% of women affected compared with 12.4% of men [184]. The impact of ovarian aging on kidney function is further underscored by studies linking early menopause to an elevated CKD risk. A study involving 4945 postmenopausal women found that women experiencing early menopause before the age of 45 years had an increased risk of CKD compared with women experiencing natural menopause at the age of ≥45 years (OR = 1.26, 95% CI = 1.01–1.56) [185]. Similarly, a population‐based study of 1653 women demonstrated a higher risk of CKD in women with bilateral oophorectomy (HR = 1.42, 95% CI = 1.14–1.77) [6]. In addition, aging‐related decline in renal function is associated with renal fibrosis, a hallmark of progressive CKD. While glomerular loss and renal fibrosis due to hypertension and diabetes progress more slowly in women than in men, this sex‐based advantage diminishes following menopause. Premenopausal women with diabetes are at a lower risk of developing end‐stage renal disease than age‐matched men, but this protective effect is lost with ovarian aging [217]. These observations suggest that estrogen supplementation may hold potential as a therapeutic strategy for mitigating CKD progression in postmenopausal women.

#### 4.2.2 Ovarian Aging on Nonalcoholic Fatty Liver Disease

Nonalcoholic fatty liver disease (NAFLD) is the primary cause of chronic liver disease and liver transplants worldwide [218]. It progresses through four histological stages: nonalcoholic fatty liver, nonalcoholic steatohepatitis (NASH), NASH cirrhosis, and NASH‐associated hepatocellular carcinoma [219]. Sex differences in NAFLD pathophysiology highlight the protective role of estrogen. Female mice demonstrate greater insulin sensitivity, enhanced fatty acid oxidation, and higher expression of c‐Jun N‐terminal kinase and AMPK compared with males [220]. Additionally, females exhibit increased hepatic expression of CD36, promoting fatty acid uptake, while a more pronounced downregulation of aquaporin 9 lowers glycerol absorption, collectively reducing liver hypertrophy [220]. Estrogen exerts multifaceted metabolic effects that mitigate NAFLD risk. It suppresses hepatic gluconeogenesis while enhancing glycogen synthesis and storage, leading to lower blood glucose levels [186]. By upregulating glucose transporter 2 and activating key glycolytic enzymes, estrogen facilitates glucose utilization and glycogen deposition [221]. It also stimulates phosphofructokinase activity and promotes glycolysis, further reducing circulating glucose levels [221]. In lipid metabolism, estrogen enhances very‐low‐density lipoprotein secretion and mitochondrial β‐oxidation, while reducing fatty acid uptake and de novo lipogenesis [222]. Additionally, it inhibits lipolysis in adipose tissue, thereby limiting the release of free fatty acids into circulation and promoting their oxidation in skeletal muscle [223]. These mechanisms collectively prevent hepatic lipid accumulation and NAFLD progression. It also lowers low‐density lipoprotein cholesterol and increases high‐density lipoprotein (HDL) cholesterol levels [187]. In estrogen‐deficient animals, cholesterol synthesis does not increase, but the conversion of cholesterol to bile acids decreases due to reduced cholesterol 7α‐hydroxylase activity, resulting in lower HDL cholesterol excretion from the liver [187]. Evidence from aged ovariectomized rodent models indicates that ovarian insufficiency exacerbates OS and hepatic inflammation, accelerating liver damage [188]. The decline in estrogen during menopause, coupled with a relative rise in androgen levels, creates a metabolic profile that predisposes individuals to atherosclerosis and insulin resistance, potentially explaining the greater prevalence of NAFLD in postmenopausal women [224].

Clinical studies support the link between ovarian aging and NAFLD risk. Estrogen deficiency, particularly following oophorectomy or POF, is associated with a heightened likelihood of NAFLD, with the risk increasing with prolonged deficiency [225, 226]. NAFLD prevalence rises substantially from 42.9% in premenopausal obese women to 60.2% in postmenopausal counterparts [190]. Another study found a 60% prevalence of NAFLD in postmenopausal women compared with 32% in premenopausal women [189]. Women with premature menopause experience a higher risk of severe liver fibrosis, and those with NAFLD have an increased risk of fibrosis progression as they age [227]. Postmenopausal women also display greater fibrosis severity, particularly in cases of hepatocellular ballooning and portal vein inflammation [228]. Menopause‐associated fat redistribution exacerbates insulin resistance, dyslipidemia, hypertension, and diabetes, further compounding NAFLD risk [229]. Hormone replacement therapy in postmenopausal women may lower the risk of NAFLD and fibrosis progression.

#### 4.2.3 Ovarian Aging on Type 2 Diabetes Mellitus

Type 2 diabetes mellitus (T2DM) has reached pandemic proportions, affecting 10.5% of the global population, with prevalence driven largely by aging and escalating obesity rates [230]. Increased obesity rates and estrogen deficiency due to ovarian aging heighten women's susceptibility to T2DM [192]. Menopausal estrogen deficiency triggers metabolic changes that lead to adiposity accumulation and redistribution, particularly central adiposity [192]. This excess adipose tissue promotes a proinflammatory milieu characterized by the release of cytokines and adipokines, which contribute to peripheral insulin resistance and chronic low‐grade systemic inflammation [191]. Beyond its effects on adipose tissue, estrogen deficiency disrupts insulin homeostasis through multiple mechanisms. It modulates insulin secretion, signaling, and degradation, thereby influencing glucose metabolism [192]. The decline in estrogen leads to increased circulating free fatty acids, elevated triglyceride levels, and impaired insulin signaling, all of which contribute to reduced insulin sensitivity [192]. Concurrently, alterations in pancreatic β‐cell function, including increased apoptosis, diminished insulin secretion, and enhanced insulin degradation, result in progressive β‐cell failure. As a consequence, the availability of free insulin declines over time, ultimately impairing glucose regulation and predisposing women to T2DM [192].

Compelling evidence from human studies also demonstrated the association between ovarian aging and T2DM. An analysis of 1407 women aged 42–52 years from the Study of Women's Health Across the Nation showed that lower estradiol levels during premenopausal and early MT periods were related to increased risk of T2DM [231]. Similarly, an 11‐year prospective study demonstrated that women with POF faced a 32% higher risk of T2DM compared with those with normal ovarian function. Additionally, a shorter reproductive lifespan correlated with a higher T2DM risk (HR = 1.06, 95% CI = 1.01–1.12) [193]. Findings from the Women's Health Initiative, which included 124,379 postmenopausal women, further substantiated this association—those with a reproductive lifespan of less than 30 years exhibited a 37% greater risk of T2DM compared with women with a reproductive span of 36–40 years [194]. A systematic review confirmed these trends, demonstrating that each year of reproductive lifespan reduction was associated with a 6–15% increase in T2DM risk [195].

#### 4.2.4 Ovarian Aging on COPD

COPD is a progressive lung disease marked by age‐related decline in lung function, partially reversible airflow obstruction, reduced lung elasticity, and alveolar enlargement [232, 233]. Emerging evidence suggests that ovarian aging and associated hormonal fluctuations influence lung structure and function. Herring et al. [196] reported a significant reduction in alveolar number in ovariectomized animals, underscoring the detrimental impact of reproductive hormone loss on alveolar morphology [196]. Conversely, estrogen replacement therapy facilitated alveolar regeneration in ovariectomized mice, highlighting estrogen's critical role in maintaining alveolar integrity [234]. Given that small airway patency depends on the tethering effect of alveolar attachments, estrogen‐mediated preservation of alveolar architecture may mitigate lung function decline and enhance respiratory capacity [197]. OS is a key contributor to COPD [198]. In a murine COPD model, progesterone treatment reduced inflammatory cell infiltration in bronchoalveolar lavage fluid, enhanced antioxidant enzyme activity, and lowered levels of malondialdehyde, a marker of lipid peroxidation, indicating its protective role against OS and inflammation in COPD [235].

Clinical studies further support the link between ovarian aging and pulmonary function decline. Postmenopausal women demonstrate poorer lung function than their premenopausal counterparts, evidenced by lower force expiratory volume in 1 s (FEV_1_) and forced vital capacity (FVC) [236]. A 21‐year cohort study involving 2020 women found that postmenopausal women experienced reduced FVC, FEV_1_, and 25–75% forced expiratory flow (FEF_25–75_) [199]. Consistently, data from the UK Biobank, encompassing 141,076 women, revealed that naturally menopausal women had diminished FVC and FEV_1_, along with an increased risk of pulmonary impairment (FVC < lower limit of normal), compared with menstruating women [200]. Notably, earlier onset of natural menopause correlated with greater declines in lung function [200]. A large prospective cohort study in the UK (2006–2010) involving 271,271 women identified an elevated risk of COPD‐related hospitalization or mortality among those who experienced menopause before the age of 47 years [201]. Similarly, an analysis of 11,258 Australian women found an inverse relationship between age at menopause and COPD risk [202]. More recent multinational research from Australia, the UK, and Sweden further reinforced the association between earlier menopause and increased COPD susceptibility [8].

#### 4.2.5 Ovarian Aging on Other Age‐Related Diseases

Beyond its impact on the aforementioned conditions, ovarian aging influences a spectrum of other age‐related diseases. Estrogen deficiency contributes to muscle atrophy and the progressive decline in muscle mass, a hallmark of age‐related sarcopenia, through mechanisms involving altered protein turnover, increased apoptosis, and enhanced protein degradation [237]. Additionally, postmenopausal estrogen depletion doubles the prevalence of age‐related macular degeneration in older women compared with men, heightening the risk of severe visual impairment [238]. Estrogen supports the health of sensory cells by promoting neuronal survival and enhancing the secretion of VEGF in the cochlea, suggesting its role in age‐related hearing loss [239, 240, 241]. The influence of estrogen extends to skin and hair health, as ERs are widely expressed in cutaneous tissues, including the scalp. Menopause‐associated collagen depletion is particularly pronounced, with levels dropping by approximately 30% within the first 5 years and continuing to decline at a rate of 2% per year over the subsequent 15 years [242]. This decline contributes to common issues like skin aging and hair loss commonly observed during MT. Moreover, declining estrogen levels compromise immune function, predisposing postmenopausal women to a higher incidence of infections and autoimmune diseases. Estrogen serves as a key regulator of humoral immunity, and its absence impairs immune homeostasis [243, 244]. Consequently, menopausal women exhibit increased susceptibility to infections such as human papillomavirus and human immunodeficiency virus and often experience exacerbations of pre‐existing autoimmune conditions [245, 246, 247].

In conclusion, ovarian aging exerts far‐reaching systemic consequences, extending beyond reduced fertility and menopausal transition to contribute to the pathogenesis of various age‐related diseases including CVD, AD, PD, and COPD. A deeper understanding of the mechanisms driving ovarian aging is crucial for recognizing its multisystem implications, and interventions aimed at delaying ovarian aging may offer promising strategies for mitigating a spectrum of age‐related organ pathologies.

## 5 Biomarkers and Diagnostic Tools

Ovarian aging is a multifactorial process characterized by the progressive decline in both the quantity and quality of follicles, compromising both reproductive capacity and systemic health. Its assessment relies on physiological, endocrine, and molecular biomarkers that reflect ovarian reserve, hormonal dynamics, and cellular integrity. Key clinical indicators including antral follicle count (AFC) and ovarian volume (OV), alongside endocrine markers such as AMH, FSH, and inhibin B, provide complementary information. Emerging molecular biomarkers, including mitochondrial metabolites, coding RNAs (e.g., microRNA [miRNA], circular RNA [circRNA]), and protein signatures, offer novel insights for early detection and mechanistic elucidation. Advanced diagnostic approaches, including multivariate models, whole‐exome sequencing (WES), and imaging‐based tools, further enhance predictive precision.

### 5.1 Biomarkers of Ovarian Ageing

#### 5.1.1 Physiological Biomarkers

Ovarian ageing reflects a progressive decline in ovarian reserve, defined by the number of PGCs. As discussed earlier, both the quantity and quality of primordial follicles diminish with age, and once the follicle pool falls below a critical threshold, ovulation ceases and menopause ensues.

AFC measured by ultrasound is a widely used noninvasive marker of ovarian reserve [248]. Typically defined as follicles 2–10 mm in diameter, AFC correlates with the primordial follicle pool, providing a theoretical basis for its use as a surrogate biomarker [249]. However, follicle size‐specific dynamics complicate this relationship. In a cross‐sectional study of 366 women aged 21–41 years, the number of 2–4 and 5–7 mm follicles declined with age, whereas the number of 8–10 mm follicles paradoxically increased [250]. Although limited, evidence suggests that AFC is low at birth (2–5 follicles), peaks around the age of 15 years (10–25 follicles), and subsequently declines [251]. Overall, AFC exhibits a negative association with age, supporting its utility as a biomarker of ovarian ageing [252].

OV, also assessed by ultrasound, is another physiological marker. In a cohort of 100 infertile women, OV demonstrated a strong negative correlation with age (*r* = −0.876) [253] In addition to chronological ageing, factors such as contraceptive use, menopausal status, and obesity also modulate OV, with menopausal status exerting the greatest influence [254, 255]. On average, OV measures 4.9 mL in premenopausal women and 2.2 mL in postmenopausal women [256]. Combining OV with counts of nongrowing follicles has been proposed to refine estimates of ovarian reserve in healthy women [251].

#### 5.1.2 Endocrine Biomarkers

AMH, secreted by GCs of pre‐antral and early antral follicles, is widely considered the most sensitive endocrine biomarker of ovarian reserve. AMH production ceases once follicle diameter exceeds 6–8 mm [252]. Unlike FSH or AFC, AMH levels remain relatively stable across the menstrual cycle, making it particularly useful for clinical assessment [252, 257]. Studies confirm that AMH outperforms age, AFC, OV, FSH, and inhibin B as a predictor of menopause [258, 259]. Even after adjusting for demographic factors, AMH is independently associated with menopausal timing and correlates with diverse reproductive outcomes, including infertility, irregular menstruation, and oligomenorrhea [260]. AMH also predicts live birth rates after IVF/intracytoplasmic sperm injection (ICSI) and the risk of diminished ovarian reserve (DOR) following chemotherapy [261, 262]. However, the lack of standardized assays remains a major limitation. While AMH can help identify women at risk of POI, it cannot precisely predict the age of onset [263].

FSH levels rise during perimenopause as declining estrogen reduces negative feedback to the hypothalamic–pituitary axis. However, FSH alone is an imperfect biomarker, as premature elevation can be masked by estradiol‐driven feedback [264]. Inhibin B, secreted by GCs in developing follicles, is also one of the factors that contribute to women of reproductive age maintain lower FSH levels [265]. As POI progresses, inhibin levels decrease significantly [266]. Studies suggest that a reduction in inhibin B levels can effectively reflect a decreased ovarian reserve in healthy reproductive‐age women, and when AFC is less than 5–7, inhibin B is more advantageous than FSH [265]. However, other studies indicate that, compared with AMH and FSH, inhibin B is less significant in terms of age‐related changes (*p *= 0.18, *p *< 0.001, and *p *= 0.12, respectively) [267].

#### 5.1.3 Emerging Biomarkers

Advances in ovarian biology continue to expand the repertoire of potential biomarkers. Mitochondrial dysfunction and energy metabolism are central to ovarian ageing, with metabolites such as coenzyme Q10 (CoQ10) and phosphatidic acid emerging as candidate biomarkers [268]. Reduced mtDNA copy number has been linked to earlier menopause, while animal studies suggest that circulating NUSN4 gene expression may predict ovarian ageing [269]. Proteostasis regulators such as cohesins SA1/SA2, deacetylase glycerol‐3‐phosphate dehydrogenase 1‐like, and plasminogen activator inhibitor 1, front fork transcription factor 3 (FOXO3) have also been implicated [270, 271, 272, 273]. Additionally, animal experiments have shown that bone morphogenetic protein 15, growth differentiation factor 9, C‐KIT, and a protein network composed of cytochrome P450 family members Fdx1, Cyp17a1, Cyp11a1, and Cyp2u1 may represent a novel biomarker for mouse with POF [274, 275].

Noncoding RNAs represent another frontier. Alterations in ovarian miRNA profiles accompany aging [276]. The miR‐200 family is elevated in murine serum exosomes [277], while miR‐146a upregulation has been observed in patients with POI [278]. In women with DOR, GC miR‐6881‐3p correlates positively with FSH and negatively with AMH and AFC [279]. circRNAs, interacting with miRNAs, modulate follicle development and hormone production, further expanding the potential biomarker landscape [275]. However, most candidates remain unvalidated in large clinical cohorts, and their translational feasibility requires further investigation.

### 5.2 Diagnostic Tool of Ovarian Aging

Current evidence‐based guidelines diagnose POI in women under the age of 40 years presenting with menstrual irregularities >4 months and elevated FSH (>25 IU/L). AMH serves as an adjunct biomarker, but interpretation requires clinical context [280]. Beyond conventional markers, novel models are being developed to improve diagnostic precision. Researchers have used nontargeted metabolomics, combined with bioinformatics, weighted gene coexpression network analysis, and machine learning (ML), to develop an XGBoost diagnostic model [268]. Since there is currently no internationally standardized testing protocol for AMH, researchers defined DOR postchemotherapy as AMH <1 ng/mL (Beckman Coulter Gen II assay) or <1770 pg/mL (Ansh picoAMH assay) at 8–24 months postchemotherapy. Combined with age, cancer type, alkylating agent exposure, and baseline AMH, a multivariate logistic regression model was established to predict DOR following chemotherapy (area under the receiver operating characteristic curve = 0.89 [95% CI 0.83–0.95]) [262].

Emerging technologies aim to enable earlier and less invasive diagnosis. WES has identified pathogenic mutations in POI patients, highlighting its utility in uncovering genetic etiologies [281]. Imaging advances, such as shear‐wave elastography, allow dynamic monitoring of ovarian fibrosis, a hallmark of aging [282]. Moreover, artificial intelligence (AI) approaches have revealed correlations between retinal age gap and AMH levels. A deep learning model (Frozen and Learning Ensemble Crossover) now enables noninvasive retinal‐based prediction of reproductive health [283].

In conclusion, combining traditional and emerging biomarkers improves the assessment of ovarian aging, supporting early intervention and personalized fertility management. Ongoing exploration of molecular and technological advances is essential to enhance diagnostic precision and biological understanding of ovarian senescence.

### 5.3 Multiomics Integration and AI‐Driven Prediction of Ovarian Aging

The biological complexity of ovarian aging extends beyond single molecular pathways, encompassing coordinated alterations in gene expression, epigenetic regulation, protein homeostasis, metabolism, and intercellular communication. Multiomics technologies, including genomics, epigenomics, transcriptomics, proteomics, metabolomics, and microbiomics, now offer unprecedented resolution for mapping these changes across cellular contexts and temporal scales [284]. For example, single‐cell RNA sequencing and spatial transcriptomics analyses of human ovarian samples have revealed transcriptional downregulation of FOXP1 and age‐associated remodeling of ECM and inflammatory pathways, highlighting potential molecular targets for intervention [285]. Similarly, integrated transcriptomic and proteomic analyses of human ovarian tissue have identified age‐related alterations in cytokine signaling, hypoxic responses, angiogenesis, ECM dynamics, intercellular communication, and regulatory networks [286]. Moreover, comparative transcriptomic and proteomic profiling of GV oocytes from young and aged mice demonstrates a marked reduction in translational efficiency, particularly among highly expressed genes, indicating a decline in oocyte quality [287]. In addition, integrated analyses combining human GC transcriptomics and methylomics with serum metabolomics have uncovered metabolic dysregulation, such as elevated acetoacetate and arachidonate levels, which reduce NAD⁺ and ATP availability while increasing OS, collectively contributing to POI [288]. Beyond biomarker discovery, multiomics integration enables multidimensional assessment of ovarian aging. By combining established clinical indicators such as AMH levels, AFC, and menopause timing with multiomics data, this integrative approach may facilitate earlier detection and more accurate prediction of ovarian aging, thereby advancing personalized fertility preservation strategies.

The analytical challenges posed by high‐dimensional multiomics datasets have accelerated the adoption of AI and ML techniques in reproductive aging research [289]. AI‐driven models can capture complex nonlinear relationships among heterogeneous biomarker sets, achieving superior predictive accuracy for ovarian reserve and reproductive lifespan compared with traditional statistical approaches. Recent studies employing ensemble ML algorithms, such as XGBoost and Light Gradient Boosting Machine (LightGBM), which integrate clinical, metabolomic, and transcriptomic parameters have demonstrated enhanced predictive performance relative to single‐biomarker models [268, 290]. Furthermore, deep learning frameworks such as OmiEmbed provide scalable solutions for integrating heterogeneous omics layers, enabling the discovery of latent biological features predictive of ovarian function [291].

Looking ahead, the convergence of multiomics profiling, AI, and large‐scale biobank data is poised to transform ovarian aging research from a descriptive to a predictive and personalized discipline. Future efforts should focus on establishing standardized longitudinal multiomics cohorts, harmonizing analytical platforms, and embedding interpretability and fairness into AI models to ensure their clinical reliability and translational applicability.

## 6 Therapeutic Interventions

Advances in understanding ovarian aging have stimulated the development of pharmacological interventions targeting this process. While hormone replacement therapy effectively alleviates estrogen deficiency‐related symptoms [292], its long‐term application is associated with potential cancer risks [293] and does not address the fundamental aging mechanisms. Thus, there remains a pressing need to develop novel and reliable therapeutic strategies capable of restoring ovarian function. Current approaches include antiaging compounds, stem cell‐based therapies, ovarian tissue transplantation (OTT), as well as innovative modalities such as tissue engineering, bioactive scaffolds, and exosomes/extracellular vesicles (EVs)‐based and miRNA therapies (Table 2). Preclinical and clinical evaluations of these treatments are systematically summarized in Table 3.

**TABLE 2:** Drugs and key targets for ovarian aging treatment.

Therapy intervention	Targets	Major effects	Species	Year	References
Antiaging drugs					
Resveratrol	STIR1	Extend telomere length and enhance telomerase activity	Mice	2013	[294]
	PI3K/Akt/mTOR signaling pathway	Attenuate oxidative stress and inhibits granulosa cell apoptosis	Rats	2018	[295]
	Caspase‐3 and PARP1	Reduce follicular DNA damage and apoptosis	Rats	2019	[296]
	Caspase‐3 and AMH	Reduce DNA damage and apoptosis in growing follicles, enhance AMH expression	Mice	2023	[297]
NAD and precursors	Mitochondria	Enhance ovarian reserve mitochondrial function and cellular energy metabolism and reduce ovarian inflammation	Mice	2020	[298]
			Mice	2022	[299]
	SIRT2	Stabilize meiotic spindle assembly and reduce aneuploidy incidence	Mice	2020	[300]
			Mice	2018	[301]
Melatonin	Sirt2/Sod2 MT1/p53 and MT/AMPK pathway	Reduce OS, meiotic failure and aneuploid, enhance oocyte viability	Mice	2020	[302]
		Reduce OS, meiotic failure and aneuploid, enhance oocyte viability	Mice	2019	[303]
	PTEN/PI3K/Akt/mTOR/AMPK pathway	Suppress inflammation, reduce apoptosis, and maintain telomere integrity.	Rats	2022	[304]
	SIRT pathway	Suppress inflammation, reduce apoptosis, and maintain telomere integrity.	Mice	2017	[305]
CoQ10	Bcl2	Suppress apoptosis and reduce OS	Mice	2023	[306]
	FSHR and PCNA	Promote folliculogenesis	Mice	2019	[307]
	Ovarian stem cells	Improve ovarian function and oocyte quality	Mice	2021	[308]
	Mitochondria	Restore mitochondrial gene expression in oocytes and improve mitochondrial activity	Mice	2015	[309]
NAC	NOX4, p22,	Reduce OS	Rats	2020	[310]
	p53, caspase‐3	Inhibit apoptosis	Rats	2020	[310]
	Telomere	Extend telomere length and increased ovarian telomerase activity	Mice	2012	[311]
Metformin	SITR1	Reduce oxidative damage	Mice	2019	[312]
	PI3K–Akt–FOXO3a	Inhibit apoptosis	Mice	2024	[313]
	AMPK	Reduce oxidative stress and inflammation	Mice	2024	[314]
	SASP fibroblasts, macrophages	Attenuate age‐related fibrosis	Mice	2022	[315]
	CD8^+^T‐cell	Reduce ovarian fibrosis	Human	2020	[316]
Stem cell therapy					
hUCMSCs	VEGFA/PI3K/AKT/mTOR	Reduce apoptosis and autophagy	Rats	2023	[317]
	Hippo signaling	Attenuate apoptosis and oxidative stress	Mice	2021	[318]
	miR‐17‐5p/SIRT7	Alleviate ROS accumulation	Mice	2020	[319]
	Nrf2/GPX4	Attenuate ferroptosis and oxidative stress	Mice	2024	[320]
	PI3K pathway	Promote the level of free amino acids, improve lipid metabolism, reduce the concentration of monosaccharides, and restore ovarian function	Mice	2020	[321]
	Change several pathways	Reduce granulosa cell apoptosis and restore ovary functionality	Mice	2013	[322]
	Secret HGF, VEGF, and IGF‐1	Improve ovarian reserve function and withstand ovarian senescence	Rats	2017	[323]
hBM‐MSCs	CYP19A1/StAR	Reduce granulosa cell apoptosis, enhance estradiol secretion, and increase follicle counts	Mice	2021	[324]
hAMSCs	Ampk/FoxO3a	Repress apoptosis	Mice	2021	[325]
BM‐MSCs	Secret VEGF	Increase the number of follicles	Rabbits	2013	[326]
**OCT/OTT**					
Needle puncturing of thawed human ovarian tissue prior to transplantation	Affect the expression of angiogenic genes	Rise follicle survival slightly but not able to increase revascularization on the human ovarian xenografts	Mice	2023	[327]
Cotransplantation of hypoxia‐preconditioned hUCMSCs with ovarian tissue	HIF1α/VEGFA signal pathway	Reduce apoptosis, increase follicle survival, and improve earlier vascularization of ovarian grafts in the early postgrafting period	Mice	2022	[328]
Frozen‐thawed human ovarian cortex pieces	/	Establish equally efficient revascularization in host from both sides of transplanted human ovarian cortex	Mice	2022	[329]
Tissue engineering and bioactive materials					
3D‐printed microporous hydrogel scaffolds	/	Support follicle development, angiogenesis, and partial fertility restoration	Mice	2017	[330]
A human decellularized ovarian scaffold based on a sodium lauryl ester sulfate (SLES)‐treated process	/	Increase estradiol and progesterone secretion	Rats	2018	[331]
**Drug delivery**					
Platelet‐rich plasma	/	Promote paracrine and vascular repair, reduce follicular atresia and inflammatory response	Rats	2020	[332]
MSC‐EVs	MSC‐derived exosomes activate Nrf2/GPX4 and PI3K/AKT cascades	Restore granulosa cell survival and endocrine balance	Mice	2024	[320]
	Change several pathways	Reduce granulosa cell apoptosis and restore ovary functionality	Mice	2013	[322]
	Ampk/FoxO3a	Repress apoptosis	Mice	2021	[325]
Exosomal miR‐10a	Modulate liposomal systems	Reduce apoptosis, improve follicle survival, and attenuate chemotherapy‐induced follicular atresia	Mice	2023	[333]
Targeted nanocarriers: zeolitic imidazolate frameworks‐8 (ZIF8)–GH@ZP3Ab	/	Promote angiogenesis, reduce oxidative stress and apoptosis	Mice	2025	[334]
Follicular‐fluid‐derived exosomes carrying miR‐320a‐3p	FOXQ1	Improve mitochondrial function and promote the ovarian granulosa proliferation	Mice	2024	[335]
CeNPs	Influence antioxidant activity	Mitigate oxidative stress in ovarian tissues and promote regeneration	Mice	2024	[336]
Exosomal miR‐17‐5P	miR‐17‐5p/SIRT7	Alleviate ROS accumulation	Mice	2020	[319]

**TABLE 3:** Clinical trials for ovarian aging treatment.

Therapy		Registration ID	Title	Start Date	Phase	Status	Enrollment	Results
Antiaging drugs	Resveratrol	NCT05410093	Clinical efficacy analysis of resveratrol in the treatment of primary ovarian insufficiency	2022/02/01	N/A	Unknown (last known status was recruiting)	150 (estimated)	No results posted^b^
		NCT06235294	Effects of resveratrol supplementation on oocyte quality	2024/09	N/A	Not yet recruiting	88 (estimated)	No results posted^b^
		NCT01782911	Effect of resveratrol on metabolic parameters and oocyte quality in PCOS patients undergoing IVF treatment	2013/02/01	N/A	Completed	10 (actual)	No results posted^b^
	NAD and precursors	NCT05485610	Effect of NMN (nicotinamide mononucleotide) on diminished ovarian reserve (including premature ovarian insufficiency)	2022/07/01	N/A	Recruiting	200 (estimated)	No results posted^b^
		NCT06426355	The efficiency of NMN in improving IVF/ICSI‐ET pregnancy outcomes in patients with DOR	2023/10/01	N/A	Recruiting	200 (estimated)	No results posted^b^
	Melatonin	IRCT2014041417264N1	Effect of melatonin on the outcome of assisted reproductive technique cycles in women with diminished ovarian reserve		N/A	Completed	66 (actual)	Increase the yield of mature MII oocytes and Grade I embryos [337]
		NCT02993588	Impact of melatonin on IVF/ICSI outcomes in prospective poor responders	2016/12	Phase 2 Phase 3	Unknown (last known status was recruiting)	100 (estimated)	No results posted^b^
		NCT03117725	Melatonin study between diminished and normal responder in IVF	2017/05/12	N/A	Unknown (last known status was enrolling by invitation)	100 (estimated)	No results posted^b^
	CoQ10	ChiCTR‐IPR‐17010945	The clinical study of patients with poor ovarian response and premature ovarian insufficiency	2015/06/02	Phase 4	Completed	169 (actual)	Improves IVF outcomes [338]
		NCT01048385	The effect of Co enzyme Q10 together with fertility drugs on pregnancy outcome of in vitro fertilization (CoQ10‐IVF)	2009/12	N/A	Terminated	34 (actual)	No results posted^b^
		NCT02010164	CoQ10 treatment to improve fertility in elderly patients	2016/12	N/A	Unknown (last known status was recruiting)	100 (estimated)	No results posted^b^
		NCT04302532	Coenzyme Q10 and fertility outcome in women with clomiphene resistant PCOS	2020/07/01	Phase 4	Completed	149 (actual)	No results posted^b^
	NAC	ChiCTR2100048297	The effect and mechanism of N‐acetylcysteine in improving in IVF in women of older age	2021/08/01	N/A	Completed	189 (actual)	Improve ovarian response to ovulation‐inducing drugs and blastocyst quality [339]
	Metformin^a^	/	/	/	/	/	/	/
Stem cell therapy		NCT03069209	Autologous bone marrow‐derived stem cell transplantation in patients with premature ovarian failure (POF)	2015/01	Phase 1 Phase 2	Unknown (last known status was active, not recruiting)	50 (estimated)	No results posted^b^
		NCT02151890	Pregnancy after stem cell transplantation in premature ovarian failure (POF)	2012/03	Phase 1 Phase 2	Completed	10 (actual)	No results posted^b^
		NCT02043743	Autologous stem cells transplantation in patients with idiopathic and drug induced premature ovarian failure	2014/01	Phase 1 Phase 2	Unknown (last known status was recruiting)	60 (estimated)	No results posted^b^
		NCT02372474	“It is a Real” the first baby of autologous stem cell therapy in premature ovarian failure	2012/03	Phase 1 Phase 2	Completed	112 (actual)	No results posted^b^
		NCT06132542	Autologous ADMSC transplantation in patients with POI (ADMSC)	2024/01/15	Phase 1	Not yet recruiting	10 (estimated)	No results posted^b^
		NCT02062931	Autologous mesenchymal stem cells transplantation in women with premature ovarian failure	2012/03	Phase 1 Phase 2	Unknown (last known status was recruiting)	60 (estimated)	No results posted^b^
		NCT02603744	Autologous adipose‐derived mesenchymal stromal cells transplantation in women with premature ovarian failure (POF)	2015/06	Phase 1 Phase 2	Unknown (last known status was recruiting)	9 (estimated)	No results posted^b^
		NCT06578039	Phase 1 clinical trial of CordSTEM‐ST (CBT210‐POI_P1)	2024/08/30	Phase 1	Completed	6 (actual)	No results posted^b^
		NCT03877471	Mesenchymal stem cells (MSCs)‐like cell transplantation in women with primary ovarian insufficiency (MSCLCTWPOI)	2019/04/03	Phase 1	Unknown (last known status was active, no recruiting)	28 (actual)	No results posted^b^
		NCT04009473	Stem cell therapy and growth factor ovarian in vitro activation (SEGOVA)	2019/06/01	Phase 1 Phase 2	Unknown (last known status was enrolling by invitation)	50 (actual)	Boost follicle development and estrogen [340]
		NCT01742533	Stem cell therapy combined hormone replacement therapy in patients with premature ovarian failure	2012/03	Phase 1 Phase 2	Unknown (last known status was recruiting)	40 (estimated)	No results posted^b^
		NCT03816852	The safety and efficiency study of mesenchymal stem cell (19#iSCLife‐POI) in premature ovarian insufficiency	2018/10/01	Phase 2	Suspended	12 (estimated)	No results posted^b^
		NCT03535480	Autologous bone marrow stem cell ovarian transplantation to restore ovarian function in premature ovarian failure (ASCOT‐2)	2018/06	Phase 4	Unknown (last known status was not yet recruiting)	20 (estimated)	No results posted^b^
		NCT05308342	Clinical study of human umbilical cord mesenchymal stem cells in the treatment of premature ovarian insufficiency	2019/11/20	N/A	Unknown (last known status was recruiting)	66 (estimated)	No results posted^b^
		NCT05138367	Effects of UCA‐PSCs in women with POF	2018/12/01	Phase 1	Completed	20 (actual)	No results posted^b^
		NCT07115082	Clinical study on the safety and efficacy of human amniotic mesenchymal stem cells in the treatment of premature ovarian insufficiency	2024/10/15	Phase 1 Phase 2	Recruiting	50 (estimated)	No results posted^b^
		NCT01853501	Effects of ADSC therapy in women with POF	2012/09	Phase 4	Unknown (last known status was enrolling by invitation)	4 (estimated)	Results submitted—not posted on ClinicalTrials.gov
		NCT03985462	Very small embryonic‐like stem cells for ovary	2019/07/03	Phase 1 Phase 2	Withdrawn	0 (actual)	No results posted^b^
		NCT02696889	Rejuvenation of premature ovarian failure with stem cells (ROSE‐1)	2016/02/06	N/A	Completed	3 (actual)	Improve premature ovarian failure symptoms significantly [341]
		NCT04675970	Long term follow up patients with premature ovarian failure ex vivo gene therapy (UB‐OVF)	2020/09/01	Not mentioned	Active, not recruiting	86 (actual)	No results posted
		NCT04475744	4‐step ASCOT in POI women to promote follicular rescue	2021/03/05	Phase 3	Completed	42 (actual)	No results posted^b^
		NCT02644447	Transplantation of HUC‐MSCs with injectable collagen scaffold for POF	2015/10	Phase 1 Phase 2	Completed	23 (actual)	No results posted^b^
		NCT02779374	Autologous bone marrow transplantation for premature ovarian insufficiency (BMT‐POI)	2016/07	N/A	Terminated	10 (estimated)	No results posted^b^
		NCT06072794	A proof of concept study to evaluate exosomes from human mesenchymal stem cells in women with premature ovarian insufficiency (POI) (VL‐POI‐01)	2023/10/06	Phase 1	Suspended	9 (estimated)	No results posted^b^
		NCT03033277	Human umbilical cord mesenchymal stem cells (HUC‐MSCs) transplantation in women with primary ovarian insufficiency (POI)	2016/02	Phase 1 Phase 2	Unknown (last known status was recruiting)	320 (estimated)	No results posted^b^
		NCT06841328	Fertility enhancement through regenerative treatment in ovaries and testes (fertile)	2025/04/08	Not mentioned	Recruiting	60 (estimated)	No results posted^b^
		NCT05494723	Safety and efficacy of YB‐1113 in treatment of POI	2026/01/09	Phase 1	Not yet recruiting	6 (estimated)	No results posted^b^
		NCT04706312	Transplantation of hAMSCs for woman with DOR	2021/04	Phase 1	Unknown (last known status was not yet recruiting)	12 (estimated)	No results posted^b^
		NCT02912104	A therapeutic trial of human amniotic epithelial cells transplantation for primary ovarian failure (POF)	2020/06/20	Phase 1	Completed	36 (actual)	Improve endometrial thickness and ovarian volume, balance hormone levels, and alleviate menopausal symptoms [342]
OTC/OTT		NCT03496636	Autologous ovarian tissue transplantation	2021/03/01	N/A	Recruiting	5 (estimated)	No results posted^b^
		NCT06673004	Ovarian tissue allo‐transplantation	2025/10/01	N/A	Not yet recruiting	10 (estimated)	No results posted^b^
		NCT05462379	Autologous heterotopic fresh ovarian graft in woman with LACC eligible for pelvic radiotherapy treatment	2022/06/08	Phase 1 Phase 2	Recruiting	20 (estimated)	No results posted^b^
		NCT02780791	Maturation of follicles after transplantation of ovarian tissue into the pelvic wall and the ovary (Ovartrans)	2017/09/10	N/A	Terminated	1 (actual)	No results posted^b^
		NCT02322060	In vitro activation of dormant follicles for patients with primary ovarian insufficiency (IVADFPOI)	2014/09	Not mentioned	Completed	100 (estimated)	No results posted^b^
		NCT01558544	Cryopreservation of ovarian tissue	1997/04	N/A	Recruiting	300 (estimated)	No results posted^b^
		NCT06261658	Improving the quality of cryopreserved ovarian tissue reimplantation using platelet‐enriched autologous plasma (TESOVA2022)	2024/03/01	N/A	Recruiting	45 (estimated)	No results posted^b^
New therapies	Tissue engineering and bioactive materials	NCT03178695	Inovium ovarian rejuvenation trials	2017/06/01	Phase 1	Completed	200 (actual)	No results posted^b^
		NCT04381299	Will autologous platelet‐rich plasma able to restore ovarian function?	2021/04/25 (actual)	Not mentioned	Active, not recruiting	35 (estimated)	No results posted^b^
		NCT04797377	Autologous intraovarian platelet‐rich plasma treatment in women with poor ovarian response	2021/03/16 (actual)	N/A	Completed	66 (actual)	No results posted^b^
		NCT06497270	A 3D bioprinted hormone‐producing model for BRCA mutated patients after risk reducing surgery: the DISC‐OVARY trial (DISC‐OVARY)	2024/10/10 (actual)	N/A	Active, not recruiting	3 (estimated)	No results posted^b^
	Drug delivery	NCT06072794	A proof of concept study to evaluate exosomes from human mesenchymal stem cells in women with premature ovarian insufficiency (POI) (VL‐POI‐01)	2023/10/06 (actual)	Phase 1	Suspended	9 (estimated)	No results posted^b^
		NCT06841328	Fertility enhancement through regenerative treatment in ovaries and testes (FERTILE)	2025/04/08 (actual)	Not mentioned	Recruiting	60 (estimated)	No results posted^b^
		NCT06202547	Intraovarian injection of MSC‐EVs in idiopathic premature ovarian failure	2023/02/20 (actual)	Phase 1 Phase 2	Recruiting	10 (estimated)	No results posted^b^
		NCT06773572	Use of autologous exosomes vs. platelet growth factors to regenerate the ovary in women with infertility (Exosomas2024‐1) (Exosom2024‐1)	2024/01/03 (actual)	N/A	Completed	30 (actual)	Improve ovarian reserve parameters (FSH, LH, estradiol, AMH, and antral follicle count), leading to significantly better fertilization, frozen embryo, and positive pregnancy rates [343]

^a^ No relevant clinical studies have been registered by 2025/08/23.

^b^ *Data sources*: clinical registration website: https://clinicaltrials.gov/.

### 6.1 Antiaging Drugs

Numerous therapeutic targets for ovarian aging have been identified, including molecular regulators (e.g., Sirtuin 1 [*SIRT1*], AMPK, NAD), antioxidant genes (IDH1, NDUFB10), and transcriptional factors (FOXP1). These targets are systematically categorized by drug–target interactions (DTIs), emphasizing the polypharmacological potential of single agents to concurrently address OS, mitochondrial dysfunction, and SASP. To advance clinical translation, the following sections detail these antiovarian aging drug targets, highlighting strategies that exploit multipathway modulation.

#### 6.1.1 Resveratrol

Resveratrol (RES), a polyphenolic compound predominantly found in grapes and red wine, exerts potent anti‐inflammatory and antioxidant effects [344]. A key activator of the longevity‐associated gene *SIRT1*, RES has been shown to enhance telomerase activity, extend telomere length, and preserve the follicular reserve in aged mice, thereby mitigating age‐related infertility [294]. In a rat model of POI, RES attenuates OS and inhibits GC apoptosis via activation of the PI3K/Akt/mTOR signaling pathway [295]. Moreover, RES demonstrates protective effects against chemotherapy‐induced ovarian damage. Preclinical studies suggest that RES reduces follicular DNA damage and apoptosis, resulting in elevated AMH levels [296, 297, 345]. These protective effects may stem from the suppression of chemotherapy‐induced caspase‐3 and PARP1 activation [296, 297, 345]. It has also been found that RES alone does not prevent cisplatin‐induced DNA damage and apoptosis in the ovary. Rather, its coadministration with melatonin induces SIRT1 overexpression, effectively protecting against DNA damage [346].

RES treatment reduces apoptosis in infertility female GCs under OS conditions [347]. Interestingly, its effects appear concentration‐dependent: while low doses (1 µM) suppress apoptosis, higher doses (10–50 µM) enhance OS, inhibit DNA synthesis, and promote apoptosis in human umbilical vein endothelial cells [348]. In contrast, another study demonstrated that both 1 and 10 µM RES significantly reduced apoptosis [347]. Besides, RES increased ATP production and cellular activity in human GCs and promoted mitochondrial biogenesis [349]. These findings suggest that RES may serve as a promising therapeutic agent for ovarian aging. However, its clinical application remains complex, as RES has been associated with decidualization effects in the endometrium and adverse events such as headaches, dizziness, and hepatotoxicity [350]. Further studies are necessary to optimize dosing strategies and evaluate its long‐term safety and efficacy.

#### 6.1.2 NAD and Precursors

NAD⁺ is a critical coenzyme involved in energy metabolism, redox homeostasis, and DNA repair [351]. NAD⁺ levels decline with age, contributing to the pathogenesis of multiple age‐related disorders, including ovarian dysfunction [352]. As a precursor of NAD⁺, nicotinamide mononucleotide (NMN) supplementation restores NAD⁺ levels and enhances ovarian reserve in aged mice by improving mitochondrial function, autophagy, protease activity, and energy metabolism, while reducing ovarian inflammation [298, 299]. NMN administration also ameliorates age‐related oocyte deterioration, potentially via SIRT2 overexpression, which stabilizes meiotic spindle assembly and reduces aneuploidy incidence [300, 301]. Given that proper spindle formation is ATP‐dependent, timely NMN supplementation may ensure adequate NAD⁺ availability to sustain this process [300]. Beyond aging, NMN has demonstrated a protective role against chemotherapy‐induced ovarian toxicity and female infertility. Importantly, its cytoprotective effects do not appear to interfere with the efficacy of cancer treatments, suggesting its potential for fertility preservation in oncology patients [353]. Another NAD⁺ precursor, nicotinamide riboside (NR), has been shown to enhance ovarian mitochondrial function, increase ovarian reserve, and improve live birth rates in aged mice [354].

However, despite promising preclinical findings, NAD⁺ depletion in human oocytes has been observed at different meiotic stages, with variability in precursor abundance [76]. The precise effects of NAD⁺ and its precursors on human ovarian function remain an active area of investigation. Future research should focus on identifying the most effective precursor, optimizing dosing regimens, and translating these findings into clinical applications.

#### 6.1.3 Melatonin

Melatonin, a potent free radical scavenger and antioxidant, is synthesized by the pineal gland and various peripheral tissues, including the ovaries, where it is present in follicular fluid [355, 356]. Aging‐associated depletion of follicular melatonin leads to excessive ROS accumulation, which compromises oocyte quality. By activating the Sirt1/Sod2 pathway, melatonin reduces OS and enhances oocyte viability [302]. Melatonin further alleviates OS through multiple signaling pathways, including melatonin membrane receptor 1 (MT1) /p53‐signaling pathway, MT1/AMPK pathway, and SIRT3 [303, 357, 358]. In addition to ROS neutralization, melatonin mitigates age‐ and chemotherapy‐induced follicular loss and ovarian dysfunction by suppressing inflammation, reducing apoptosis, and maintaining telomere integrity, with the PTEN/PI3K/Akt/mTOR/AMPK and SIRT pathways playing central roles [304, 305, 358, 359]. Moreover, melatonin preserves intercellular communication, a key factor in sustaining oocyte quality during aging [360].

Clinical studies further support melatonin's role in ovarian function. Women with DOR exhibit lower melatonin levels in follicular fluid and serum compared with those with normal ovarian function [361]. Serum and follicular melatonin levels negatively correlate with age and baseline FSH but positively correlate with AMH and AFC, suggesting a strong link between melatonin levels and ovarian reserve [361]. Another study found that melatonin supplementation improved in IVF outcomes in older women, with a significant increase in clinical pregnancy rates (from 20.3 to 46.0%), ongoing pregnancy rates (from 15.3 to 36.5%), and live birth rates (from 15.3 to 33.3%) [362]. Notably, animal studies indicate that the timing of melatonin intervention is critical. Initiating melatonin supplementation at 23 weeks of age in mice significantly enhances reproductive outcomes, whereas treatment beginning at 33 weeks yields no significant benefits compared with controls [363]. These findings suggest that melatonin therapy may be most effective when administered before the onset of ovarian aging. However, there are currently no corresponding population studies indicating the appropriate timing and dosage range for melatonin treatment of ovarian aging. Larger clinical studies are needed in the future.

#### 6.1.4 CoQ10‐Mitochondrial Therapy

CoQ10 is an important component involved in the electron transport chain and plays an essential role in protecting mitochondria from ROS‐induced oxidative damage [307, 364]. The CoQ10 content of oocytes progressively decreases with age, which is consistent with diminished oocyte quality and age‐related fertility decline [309]. As a potent antioxidant, CoQ10 enhances oocyte quality by reducing ROS and increasing *Bcl2* and *Sirt1* expression, indicating that it may alleviate ovarian aging by suppressing apoptosis [306, 307, 365]. A study applying cyclophosphamide (CTX)‐induced mouse POF model showed that CoQ10 acts on FSHR and proliferation cell nuclear antigen (PCNA), which are important genes related to folliculogenesis [307]. In addition, CoQ10 facilitates the differentiation of ovarian stem cells derived from the ovarian surface epithelium and improves ovarian function and oocyte quality in a mouse model of chemically induced ovarian failure [308]. Mitochondrial dysfunction is a hallmark of ovarian aging [87]. Supplementation of CoQ10 in aged mice delays ovarian reserve depletion, restores mitochondrial gene expression in oocytes, and improves mitochondrial activity [309]. Intriguingly, CoQ10 exerts no effect on ovarian reserve or oocyte quality in young females, indicating that its benefits are specific to age‐related mitochondrial dysfunction [309]. In patients undergoing IVF, decreased CoQ10 levels in granular cells have been associated with impaired mitochondrial function, while in vitro CoQ10 supplementation has been shown to enhance mitochondrial activity in these cells [366].

Clinical studies suggest a potential role for CoQ10 in improving reproductive outcomes. A randomized, double‐blind study in reproductively older women reported a reduction in oocyte aneuploidy rates with CoQ10 treatment (62.8 vs. 46.8%) and a modest increase in clinical pregnancy rates (26.7–33%), although the results did not reach statistical significance due to the limited sample size [367]. In younger women with DOR, CoQ10 supplementation significantly elevated serum estrogen levels, enhanced ovarian response to stimulation, and improved oocyte and embryo quality during IVF–ICSI cycles, leading to higher clinical pregnancy and live birth rates and a lower miscarriage rate [338]. A meta‐analysis identified an optimal regimen of 30 mg/day CoQ10 for 3 months before ovarian stimulation, particularly benefiting women with DOR under 35 years of age [368]. Although current evidence highlights CoQ10 as a promising therapeutic for ovarian aging, its impact on live birth rates remains uncertain. Further large‐scale animal and clinical studies are essential to validate the clinical efficacy and optimize the therapeutic protocol for CoQ10 in ovarian aging [368].

#### 6.1.5 N‐Acetyl‐l‐Cysteine

N‐acetyl‐L‐cysteine (NAC) has been widely used as a therapeutic agent for acetaminophen (paracetamol) overdose. As a precursor for glutathione synthesis, NAC possesses potent antioxidant properties and functions as both a direct and indirect free radical scavenger [369, 370, 371]. Studies suggest that NAC supplementation in the oocyte culture medium can counteract OS‐induced damage [371]. In addition to reducing OS by lowering NOX4 and p22 levels, NAC inhibits ovarian apoptosis by suppressing the p53/caspase‐3 cascade reaction to ameliorate radiation‐induced POF in rats [310]. Additionally, NAC has been shown to alleviate chemotherapy‐induced ovarian toxicity, improving ovarian function in treated mice [372]. Long‐term NAC administration in drinking water has been reported to delay oocyte aging and enhance oocyte quality in aged mice. Compared with controls, NAC‐treated mice exhibited increased ovarian telomerase activity and extended telomere length, both of which are indicative of improved ovarian health [311]. Despite these promising findings, studies on NAC in ovarian aging remain limited. Further investigations are necessary to determine its therapeutic efficacy and safety, particularly in clinical applications.

#### 6.1.6 Metformin

Metformin, a first‐line therapy for T2DM, is widely recognized for its favorable safety profile and high tolerability [373]. Beyond its antidiabetic properties, metformin has been implicated in reducing cancer risk, improving cancer prognosis, and delaying aging in nonhuman primates [374, 375, 376]. In aged mice, metformin treatment has been shown to mitigate ovarian aging by enhancing SIRT1 expression and reducing oxidative damage, thereby preserving ovarian reserve [312]. Studies of both murine and human ovaries suggest that metformin counteracts age‐related fibrosis by modulating the ovarian immune microenvironment and altering the balance of fibroblasts, myofibroblasts, and immune cells [315, 316]. Masson's trichrome staining revealed that while untreated postmenopausal ovaries exhibited collagen structures characteristic of age‐related fibrotic remodeling, those exposed to metformin retained a more youthful collagen organization similar to that of premenopausal ovaries [316]. Additionally, metformin has been shown to inhibit apoptosis via the PI3K–Akt–FOXO3a pathway, partially reversing d‐galactose‐induced ovarian dysfunction in mice [313].

Metformin also protects against chemotherapy‐induced ovarian damage in mice, primarily by reducing OS and inflammation, a process closely linked to its activation of the AMPK pathway [314]. In human ovaries, metformin has been shown to regulate multiple metabolic pathways associated with POI through AMPK upregulation [377]. Notably, AMPK expression negatively correlates with body mass index, FSH, and leptin levels, while positively correlating with estrogen levels in POI patients [377]. A 2‐month intervention with a therapeutic diabetic dose of metformin in nine women with POI resulted in increased AMPK activation and ATP levels, alongside decreased leptin and TNF‐α concentrations in blood mononuclear cells, with no reported adverse effects [377]. In the context of assisted reproduction, low‐dose metformin may enhance pregnancy outcomes in IVF patients without PCOS by improving insulin sensitivity [378]. However, despite its promising potential in ovarian aging, excessive metformin use can lead to lactic acidosis, particularly in patients with severe CKD, and is contraindicated in individuals with an estimated glomerular filtration rate (eGFR) below 30 mL/min [379]. Generally, metformin represents a compelling candidate for mitigating ovarian aging through both direct and indirect activation of AMPK [373]. Nevertheless, further clinical studies are essential to fully elucidate its therapeutic potential and establish its efficacy in preserving ovarian function and fertility.

Despite promising preclinical and clinical findings, several limitations hinder the therapeutic translation of antiaging drugs for ovarian aging. Melatonin and CoQ10, while improving oocyte quality and IVF outcomes, face challenges such as poor bioavailability, variability in patient response, and uncertainty regarding optimal timing and dosing, with limited evidence for long‐term safety. Similarly, NAC demonstrates antioxidative and telomere‐preserving benefits in animal models, but data remain scarce, and its clinical relevance is yet to be established. Metformin shows potential through AMPK activation and modulation of ovarian fibrosis; however, its application is constrained by risks of lactic acidosis in patients with renal impairment and limited clinical trials specific to ovarian aging. Importantly, most studies focus on monotherapies, failing to address the multifactorial mechanisms of ovarian decline. A lack of large‐scale randomized controlled trials (RCTs) and incomplete mechanistic insights further restricts their clinical adoption, emphasizing the need for multitargeted strategies and rigorous translational studies.

### 6.2 Stem Cells Therapy

Stem cell‐based therapies, particularly using mesenchymal stem cells (MSCs), have emerged as a promising strategy to counter ovarian aging and POI. MSCs, derived from bone marrow, adipose tissue, or umbilical cord, placenta, or periodontal ligament, exert their effects predominantly through paracrine mechanisms and EVs. Human MSC secretome directly stimulates GC proliferation and steroidogenesis via CYP19A1 and StAR upregulation [324]. Moreover, MSC secretomes enhance GC survival and function, reducing apoptosis and autophagy via the VEGFA–PI3K/AKT/mTOR axis [317]. MSC‐derived EVs attenuate apoptosis and OS by activating PI3K/AKT [380], modulating Hippo signaling [318], and delivering miRNAs such as miR‐17‐5p that downregulate SIRT7 [319]. MSC‐EVs also mitigate cellular damage by attenuating ferroptosis and OS, via Nrf2/GPX4 axis regulation [320]. Collectively, these actions offer a prosurvival, proproliferative, and antioxidative profile that helps preserve ovarian reserve and function.

MSC‐based interventions have been extensively validated in animal models of ovarian injury and aging, with mice and rats providing the strongest preclinical evidence. In murine chemotherapy‐induced POI models, intraovarian or systemic administration of bone marrow‐ or umbilical cord‐derived MSCs significantly restored follicle development and endocrine function. Park et al. [324] reported a rise in follicle counts after MSC secretome treatment, accompanied by enhanced estradiol secretion and reduced GC apoptosis. Similarly, Zhao et al. [321] demonstrated normalization of estrous cycles, increased AMH, and successful pregnancies in MSC‐treated mice compared with CTX‐injured counterparts. In natural ovarian aging or DOR models, MSC‐derived EVs improved primordial and growing follicle numbers, elevated AMH and estradiol, and reduced apoptotic signaling [322, 325]. Rat models corroborate these findings. When human UCMSCs were transplanted into rats at various time points, including days 14, 21, and 28, the levels of FSH decreased, while AMH and estradiol levels increased, which led to enhancements in ovarian reserve function and an increase in the number of follicles [323]. Furthermore, in a study where MSCs derived from the bone marrow of male rabbits were transplanted into a rabbit model of POF, the treatment led to a decrease in FSH levels, and an increase in the number of follicles with normal architecture [326]. Across models, common readouts include higher follicle counts, restored sex steroid profiles (↑AMH, ↑estradiol, ↓FSH), normalized cyclicity, and enhanced fertility metrics (pregnancy and litter size).

Prospective, uncontrolled or small controlled studies suggest feasibility and preliminary signals of benefit, but heterogeneity is high. The ASCOT approach (intra‐arterial bone‐marrow stem‐cell apheresis to ovaries) in poor responders reported improved oocyte yield and clinical pregnancies [381]. Intraovarian MSCs restored menses and improved ovarian function in a subset of POF patients [382], with follow‐up suggesting acceptable short‐term safety [383]. Autologous adipose‐derived stromal cells have been tested for POF in early trials (dose‐finding; feasibility) with hormonal/menstrual improvements and successful pregnancies in some participants [384, 385]. Randomized data are scarce. One small double‐blind RCTs of stromal vascular fraction for “ovarian rejuvenation” has been reported but remains exploratory and underpowered [385]. Several registered trials of MSCs are ongoing, including randomized designs.

Despite encouraging preclinical findings, MSC therapy for ovarian aging faces major translational barriers. The existing evidence is limited by low quality, with current human studies consisting predominantly of small, nonrandomized, and highly heterogeneous trials. There is a notable absence of RCTs using live birth as a primary endpoint. Furthermore, standardization is inadequate across multiple facets, with inconsistent good clinical practice protocols, cell source, potency assays, and uncertain optimal dose, timing, or delivery route. Safety is preliminarily acceptable, but long‐term risks, including ectopic growth, off‐target angiogenesis, thromboembolism, tumorigenicity, and effects on offspring, require systematic monitoring. Regulatory hurdles, variable legal frameworks, and absence of validated release criteria further complicate progress. Finally, generalizability remains unclear, since most trials target POI or poor responders, while efficacy in physiological ovarian aging is untested.

### 6.3 Ovarian Tissue Cryopreservation and Transplantation

Ovarian tissue cryopreservation (OTC) and OTT preserve the primordial follicle pool by excising and cryopreserving ovarian cortical strips, which upon transplantation can restore both endocrine activity and fertility potential. Success hinges on rapid revascularization of the graft, minimizing ischemia–reperfusion injury and consequent follicle loss [386, 387, 388]. In mice models, cryopreservation followed by orthotopic implantation restores ovarian cycles, hormone levels, and fertility [387]. Adjuncts such as antioxidants further enhance graft survival and reproductive outcomes [388]. Human evidence is now substantial. Over 200 live births have been reported worldwide following autologous OTT in cancer survivors and other iatrogenic POI populations [389]. Meta‐analyses covering hundreds of women report pooled pregnancy rates of 37%, live birth rates of 28%, and median endocrine recovery times of 19 weeks. Etiology‐adjusted median graft lifespans span 2.5 years [390]. Centers with long‐standing programs, such as Bologna University, report ovarian function restoration in about 88% of menopausal women, with four healthy live births [391]. Guidelines from ASRM and ESHRE endorse OTC as a standard fertility preservation strategy, particularly for prepubertal patients or those requiring immediate gonadotoxic treatment [392].

Currently, OTC is predominantly utilized within the context of oncofertility. Its application to physiological aging remains experimentally unvalidated and involves considerable ethical ambiguity. Risk of reintroducing malignant cells, particularly in hematologic cancers, necessitates stringent safety protocols [393]. Additionally, substantial early follicle loss resulting from ischemic damage posttransplantation continues to pose a major challenge. Technical variability in tissue processing methods, such as slow freezing versus vitrification, differences in tissue fragment size, and inconsistencies in surgical and thawing techniques, further contributes to heterogeneous outcomes across studies. Finally, long‐term maternal and neonatal health data are limited, with few registry‐driven outcome studies.

### 6.4 New Therapies

Emerging therapies for ovarian aging increasingly integrate principles of tissue engineering, nanomedicine, and molecular modulation. Tissue engineering employs decellularized ovarian scaffolds to support follicular niche reconstruction and angiogenesis [330, 394]. Bioactive hydrogels, such as silk fibroin‐based injectable matrices, support encapsulation of ovarian stromal cells and maintain viability and proliferation [395]. Drug delivery and nanocarriers, such as antioxidant nanomaterials (e.g., cerium oxide nanoparticles [CeNPs]), offer ROS‐scavenging, tissue‐protective capabilities that may preserve ovarian reserve [336]. EVs, particularly MSC‐derived exosomes, restore ovarian function through transfer of miRNAs and proteins that attenuate apoptosis and OS [319]. miRNA therapies, such as miR‐17‐5p, rescues ovarian phenotype in a chemotherapy‐induced POI mouse model via suppression of SIRT7 [319]. These techniques converge on antioxidative, proangiogenic, and antifibrotic mechanisms, offering promising cell‐free alternatives to stem cell transplantation, though evidence remains predominantly preclinical.

#### 6.4.1 Tissue Engineering and Bioactive Materials

Tissue engineering offers a promising strategy to counter ovarian aging by reconstructing the follicular microenvironment through biomimetic scaffolds. Decellularized whole‐ovarian ECM scaffolds preserve the native ultrastructure and biochemical cues necessary for follicle survival and steroidogenesis, while recellularization with homologous fibroblasts restores endocrine functionality [394]. In rats, human ovary‐derived decellularized scaffolds seeded with ovarian cells maintained follicular structures and significantly increased estradiol and progesterone secretion [331]. Similarly, ovary decellularization in mice generated bioscaffolds that sustained cell viability and recapitulated ovarian architecture [396]. Synthetic hydrogels provide an alternative, with 3D‐printed microporous constructs supporting follicle development, angiogenesis, and even partial fertility restoration in mice [330]. Despite these advances, translation to human application remains limited to in vitro models, with major barriers including ischemic follicle loss, variable scaffold preparation, and incomplete understanding of long‐term safety. Nonetheless, by integrating biomaterial science with reproductive biology, tissue engineering holds potential as a next‐generation approach to extend ovarian health span.

#### 6.4.2 Drug Delivery

Emerging drug delivery systems, stem‐cell‐derived EVs and nucleic acid therapeutics, platelet‐rich plasma (PRP), and nanocarriers, have emerged as promising targeted strategies to restore ovarian function. MSC‐derived exosomes and nanocarriers can concentrate bioactive molecules within the ovarian niche, modulating OS, apoptosis, and inflammatory pathways. Activation of Nrf2/GPX4 and PI3K/AKT cascades has been shown to restore GC survival and endocrine balance [320, 380]. In addition, miRNAs, which are key posttranscriptional regulators in folliculogenesis, oocyte maturation, and steroidogenesis [397], represent a novel frontier in therapeutics. In preclinical models, delivery of protective miRNAs, such as exosomal miR‐10a, via liposomal systems attenuates chemotherapy‐induced follicular atresia by targeting apoptotic pathways and improving follicle survival [333]. Follicular‐fluid‐derived exosomes carrying miR‐320a‐3p also rejuvenate ovarian aging through FOXQ1 inhibition [335]. PRP delivers angiogenic and mitogenic growth factors, promoting paracrine and vascular repair in situ [332, 398]. Follicle‐targeted nanocarriers encapsulating growth hormone alleviate aging‐associated dysfunction, restoring follicular activity and reducing ROS [334]. A recent study demonstrated that CeNPs, known for their catalytic antioxidant activity, significantly mitigate OS in ovarian tissues and promote regeneration in aged models, adding a novel functional nanomaterial to the ovarian rejuvenation armamentarium [336].

Rodent models of ovarian aging, induced by chemotherapy or natural senescence, demonstrate that MSC‐EVs restore follicle counts, normalize hormone profiles, and improve fertility outcomes [320, 322, 325]. Exosomal miR‐17‐5P delivery alleviated ROS accumulation and restored ovarian phenotype and function in CTX‐induced POI model mice by inhibiting SIRT7 expression [319]. Direct ovarian injection of PRP may enhance follicle development and oocyte retrieval. In a rat model of ovarian ischemia, PRP increased VEGF levels and protected against reperfusion‐related oxidative damage, likely due to its proangiogenic and proliferative properties [399]. Current clinical data remain preliminary. Meta‐analyses indicate that intraovarian PRP injections can improve ovarian reserve markers and reproductive parameters in women with DOR [400]. Stem cell therapies are in nascent trials, while miRNA‐based and nanocarrier approaches have yet to reach clinical application. CeNPs are so far confined to animal studies with no human data.

There are several limitations of the drug delivery. Translation is hindered by (i) scarcity of RCTs with live birth as primary endpoint, (ii) uncertain long‐term safety and intergenerational risks of nanoparticles and gene therapies, (iii) heterogeneity in PRP and EV preparation, and (iv) regulatory ambiguity regarding classification of biologics and nanomaterials. Standardization of biologic preparations, integration of miRNA delivery platforms with nanocarriers, and further exploration of redox‐active nanoparticles such as CeNPs are promising avenues. Long‐term safety validation, intergenerational toxicology, and rigorous multicenter trials remain critical to move from experimental promise to clinical translation.

In summary, emerging interventional strategies offer promising avenues for delay ovarian aging and extending reproductive potential. Future advances will require the integration of foundational research with translational applications, applications, ultimately bridging fertility preservation with broader goals of promoting healthy longevity.

### 6.5 Translational Challenges

Despite considerable promise, regenerative and antiaging strategies face nontrivial translational barriers before clinical adoption. Stem cell‐based therapies remain constrained by risks of tumorigenicity, genomic instability, and lineage mis‐specification, underscoring the necessity of rigorous safety screening and quality control [401, 402]. Exosome‐based interventions, although appealing as cell‐free alternatives, contend with challenges of heterogeneity, immunogenic potential, and difficulties in large‐scale production [403, 404, 405]. Tissue engineering approaches likewise must overcome hurdles of vascularization, reproducibility, manufacturing complexity, and regulatory uncertainty [406, 407, 408]. Consequently, advancing the field requires harmonized quality standards, scalable biomanufacturing, and definitive regulatory paradigms.

## 7 Conclusion and Prospects

Ovarian aging has traditionally been attributed primarily to the inevitable depletion of follicles. In this review, we reframe it as a multifaceted process arising from the interplay between intrinsic oocyte decline and dynamic alterations in the ovarian microenvironment, encompassing vascular dysfunction, stromal fibrosis, immune polarization, proteostatic collapse, and mitochondrial impairment. This integrated view positions the ovary not as a passive victim of aging but as an active regulator of systemic physiological decline. The deterioration of ovarian function exerts far‐reaching effects across endocrine and metabolic axes, influencing the pathogenesis of age‐related diseases such as AD, cardiovascular dysfunction, and osteoporosis. By synthesizing mechanistic hallmarks with translational advances, spanning biomarkers, cell‐ and nano‐based therapeutics, and rational combination strategies, this review provides a framework for repositioning ovarian aging at the interface of reproductive biology and systemic health. This perspective not only integrates diverse strands of evidence but also defines future priorities, including mechanistically informed biomarkers, combinatorial interventions, and intergenerational safety assessment of emerging therapies.

However, research on the systemic impacts of ovarian aging is limited. Studies disproportionately focus on a narrow spectrum of outcomes, such as CVD, neurocognitive disorders, metabolic changes, and osteoporosis, while evidence for broader systemic effects is sparse and of variable quality. Current research also often emphasizes the effects of estrogen deficiency, neglecting the broader systemic consequences of ovarian aging, such as inflammation and changes in other ovarian hormones like AMH. Furthermore, the effects of ovarian aging on other organs may be bidirectional, with organ interactions influencing the aging process. For example, metabolic changes associated with ovarian aging, like increased fat tissue accumulation and insulin resistance, elevate diabetes risk, while diabetes itself can influence ovarian aging [192]. Future research should adopt multidisciplinary approaches integrating hormone, inflammation, and cellular aging pathways and utilize large‐scale cohort studies to elucidate the systemic effects of ovarian aging and develop interventions to mitigate age‐related multiorgan dysfunction in women.

Therapeutic development is progressing across multiple domains. Small‐molecule and nutriceutical candidates (e.g., resveratrol, melatonin, NAD⁺ precursors, metformin) target sirtuin/AMPK redox signaling and mitochondrial quality control, with encouraging preclinical and early clinical readouts. MSC therapy shows promise as a future treatment for ovarian aging and POI, with clinical studies emerging worldwide. Where clinically indicated, OTC/OTT already delivers endocrine restoration and live births in oncofertility, providing a translational benchmark for emerging modalities. Parallel progress in tissue engineering (decellularized ovarian ECM, biomimetic hydrogels) and targeted delivery (exosomes, miRNA cargos, redox‐active nanomaterials) offers cell‐free routes to restore the follicular niche. CeNPs effectively reduce OS and promote regeneration in aging ovarian tissue, suggesting their potential as a functional nanomaterial for ovarian rejuvenation [336].

The field should address two major gaps. First, most drugs are evaluated as monotherapies, while biological mechanisms are multidimensional. Rational combination strategies, like resveratrol and melatonin targeting SIRT1‐dependent antioxidant programs and DDRs, are mechanistically sound and preclinically supported, but underexplored in human trials. Researchers found that resveratrol and melatonin together provided greater protection for pig oocyte maturation under heat stress than resveratrol alone [409]. Second, next‐generation approaches, including autophagy modulators, miRNA delivery systems, and CRISPR‐based genome repair, require robust efficacy and safety datasets, with particular attention to intergenerational effects and fibrotic remodeling. Addressing these gaps will be essential for translating mechanistic insights into interventions that extend reproductive lifespan and improve systemic health and healthy aging trajectories in women.

## Author Contributions

Conceptualization: JJZ. Writing—original draft: XYL and YQZ. Writing—review and editing: XYL, YQZ, YZF, JJZ, and SXW. Visualization: JJZ and SXW. Funding acquisition: SXW and JJZ. All authors approved the final version of the paper.

## Funding

This work was supported by the National Key Research and Development Program of China (2022YFC2704100) and the National Natural Science Foundation of China (82371648, 82471678).

## Ethics Statements

The authors have nothing to report.

## Conflicts of Interest

The authors declare no conflicts of interest.

## Supporting information




**Table S1**: The evidence linking oocyte quality indicators with ART clinical outcomes.
**Table S2**: The evidence grades of association between ovarian aging and age‐related disease based on Joanna Briggs Institute (JBI) system.

## Data Availability

The authors have nothing to report.
