# Supplementary material for: Ovarian Aging: Mechanisms, Age‐Related Disorders, and Therapeutic Interventions
Source: MedComm (2020). 2025 Nov 16;6(12):e70481. doi: 10.1002/mco2.70481 (PMC12620568; doi:10.1002/mco2.70481)
Supplement: Supplementary file 1 — Table S1: The evidence linking oocyte quality indicators with ART clinical outcomes. Table S2: The evidence grades of association between ovarian aging and age‐related disease based on Joanna Briggs Institute (JBI) system. [file MCO2-6-e70481-s001.docx]

**Ovarian Aging: Mechanisms, Age-related Disorders and Therapeutic interventions**

Xingyu Liu^1,2,3#^, Yuanqu Zhao^1,2,3#^, Yanzhi Feng^1,2,3^, Shixuan Wang^1,2,3*^, Jinjin Zhang^1,2,3^^*^

^1^ Department of Obstetrics and Gynecology, Tongji Hospital, Tongji Medical College, Huazhong University of Science and Technology, Wuhan, 430030, China

^2^ National Clinical Research Center for Obstetrical and Gynecological Diseases, Huazhong University of Science and Technology, Wuhan, 430030, China

^3^ Key Laboratory of Cancer Invasion and Metastasis, Ministry of Education, Huazhong University of Science and Technology, Wuhan, 430030, China

^#^These authors contributed equally.

*Corresponding author

Prof. Shixuan Wang, Department of Obstetrics and Gynecology, Tongji Hospital, Tongji Medical College, Huazhong University of Science and Technology, 1095 Jiefang Ave, Wuhan 430000, China. Tel: 86-27-83663351; Fax: 86-27-83662681; E-mail: [shixuanwang@tjh.tjmu.edu.cn](mailto:shixuanwang@tjh.tjmu.edu.cn) (ORCID: 0000-0002-8610-952X).

Or

Prof. Jinjin Zhang, Department of Obstetrics and Gynecology, Tongji Hospital, Tongji Medical College, Huazhong University of Science and Technology, 1095 Jiefang Ave, Wuhan 430000, China. Tel: 86-27-83663351; Fax: 86-27-83662681; E-mail: [jinjinzhang@tjh.tjmu.edu.cn](mailto:jinjinzhang@tjh.tjmu.edu.cn) (ORCID: 0000-0002-5557-3637).

**Table S1. The evidence linking oocyte quality indicators with ART clinical outcomes.**

| **Indicator** | **n (or model)** | **Key numeric outcome** | **Evidence level** | **Reference** |
| --- | --- | --- | --- | --- |
| **Zona pellucida thickness (ZPT)** | 5,184 embryos (744 IVF pts) | No predictive value for pregnancy (*P* > 0.5); “good” vs “poor” morphology embryos: 15.87 ± 2.48 µm vs 16.36 ± 2.57 µm | Large human cohort, low predictive power | ^[1]^ |
|  | 1,665 oocytes (978 cycles) | No association between ZPT and embryonic outcome | Large human cohort, negative finding | ^[2]^ |
| **Polar body morphology** | ICSI patients | Intact vs fragmented PB: implantation 48.6% vs 22.0%; ongoing pregnancy 68.4% vs 34.8% (*P* < 0.05) | Human cohort, strong predictive signal | ^[3]^ |
| **Cytoplasmic uniformity** | 1,056 MII oocytes | Higher implantation with uniform cytoplasm (≈11.1% vs 7.3%) | Moderate human cohort, moderate evidence | ^[4]^ |
| **Cytoplasmic granularity** | 2,448 MII oocytes | Refractile bodies: 12% lower cleavage, fragmentation increased | Human retrospective, consistent | ^[5]^ |
|  | 633 ICSI cycles | High CLCG: cleavage 82% vs 99%, ongoing pregnancy 14% vs 32%, live birth 13% vs 30%, miscarriage 47% vs 11% (OR 3.1, 95% CI 2.1–4.1) | Human multicenter, strong evidence | ^[6]^ |
| **Mitochondrial membrane potential (ΔΨm)** | Human oocytes (vitrification) | Vitrification reduced ΔΨm; lower ΔΨm linked to impaired competence | Small human experimental | ^[7]^ |
|  | Mouse/human models | ΔΨm dynamically enriched around meiotic spindle; higher ΔΨm linked to successful maturation | Translational, mechanistic | ^[8]^ |
|  | Human embryos | Age-related ΔΨm decline correlates with reduced developmental competence | Human observational, mechanistic | ^[9]^ |
| **Chromatin configuration** | Mouse GV oocytes | SN oocytes: 83% blastocyst vs 4% for NSN | Animal model, strong mechanistic signal | ^[10]^ |

ICSI: Intracytoplasmic sperm injection; GV, germinal vesicle; SN, surrounded nucleolus; NSN, non-surrounded nucleolus.

**Reference**

[1]. Balakier H, Sojecki A, Motamedi G, Bashar S, Mandel R, Librach C. Is the zona pellucida thickness of human embryos influenced by women's age and hormonal levels? *Fertility and sterility.* 2012;98(1):77-83.

[2]. Kawakami M, Okimura T, Uchiyama K, et al. The effect of zona pellucida thickness variation on fertilization and subsequent embryonic development: a large single center cohort study. 2019;112(3):e128-e129.

[3]. Ebner T, Moser M, Sommergruber M, Yaman C, Pfleger U, Tews G. First polar body morphology and blastocyst formation rate in ICSI patients. *Human reproduction (Oxford, England).* 2002;17(9):2415-2418.

[4]. Qassem EG, Falah KM, Aghaways IH, Salih TAJAMI. A correlative study of oocytes morphology with fertilization, cleavage, embryo quality and implantation rates after intra cytoplasmic sperm injection. 2015;2(1):7-13.

[5]. Fancsovits P, Tóthné ZG, Murber Á, Rigo Jr J, Urbancsek JJABH. Importance of cytoplasmic granularity of human oocytes in in vitro fertilization treatments. 2012;63(2):189-201.

[6]. Merviel P, Cabry R, Chardon K, et al. Impact of oocytes with CLCG on ICSI outcomes and their potential relation to pesticide exposure. *Journal of ovarian research.* 2017;10(1):42.

[7]. Chen C, Han S, Liu W, Wang Y, Huang G. Effect of vitrification on mitochondrial membrane potential in human metaphase II oocytes. *Journal of assisted reproduction and genetics.* 2012;29(10):1045-1050.

[8]. Al-Zubaidi U, Liu J, Cinar O, Robker RL, Adhikari D, Carroll J. The spatio-temporal dynamics of mitochondrial membrane potential during oocyte maturation. *Molecular human reproduction.* 2019;25(11):695-705.

[9]. Van Blerkom JJM. Mitochondrial function in the human oocyte and embryo and their role in developmental competence. 2011;11(5):797-813.

[10]. Inoue A, Nakajima R, Nagata M, Aoki F. Contribution of the oocyte nucleus and cytoplasm to the determination of meiotic and developmental competence in mice. *Human reproduction (Oxford, England).* 2008;23(6):1377-1384.

**Table S2. The evidence grades of association between ovarian aging and age-related disease based on Joanna Briggs Institute (JBI) system.**

| **Disease Type** | **Age-Related Disease** | **Type of the Study** | **Highest Evidence Grade** | **Reference** |
| --- | --- | --- | --- | --- |
| Core disease | Cardiovascular disease | - Quasi-experimental prospectively controlled study | Level 2 | ^[1]^ |
|  |  | - Systematic review of comparable cohort and other lower study design - Cohort study with control group | Level 3 | ^[2-6]^ |
|  |  | - Cross-sectional study | Level 4 | ^[7]^ |
|  |  | - Bench research | Level 5 | ^[8-12]^ |
|  | Alzheimer’s disease | - Systematic review of comparable cohort studies - Cohort study with control group - Case-controlled study | Level 3 | ^[13-16]^ |
|  |  | - Cross-sectional study | Level 4 | ^[15,17,18]^ |
|  |  | - Bench research | Level 5 | ^[19,20]^ |
|  | Parkinson’s disease | - Mendelian randomization | Level 1-2 | ^[21]^ |
|  |  | - Cohort study with control group - Case-controlled study | Level 3 | ^[22-25]^ |
|  |  | - Bench research | Level 5 | ^[26-29]^ |
|  | Osteoporosis | - Cohort study with control study | Level 3 | ^[30]^ |
|  |  | - Cross-sectional study | Level 4 | ^[31-36]^ |
|  |  | - Bench research | Level 5 | ^[37,38]^ |
| Potentially associated disease | Chronic kidney disease | - Cohort study with control group | Level 3 | ^[39,40]^ |
|  |  | - Cross-sectional study | Level 4 | ^[41,42]^ |
|  |  | - Bench research | Level 5 | ^[43-47]^ |
|  | Non-alcoholic fatty liver disease | - Cohort study with control group | Level 3 | ^[48,49]^ |
|  |  | - Cross-sectional study | Level 4 | ^[49-52]^ |
|  |  | - Bench research | Level 5 | ^[53-60]^ |
|  | Type-2diabetes mellitus | - Systematic review of comparable cohort and other lower study designs - Cohort study with control group | Level 3 | ^[61-64]^ |
|  |  | - Bench research | Level 5 | ^[65,66]^ |
|  | Chronic obstructive pulmonary disease | - Cohort study with control group | Level 3 | ^[67-70]^ |
|  |  | - Cross-sectional study | Level 4 | ^[71,72]^ |
|  |  | - Bench research | Level 5 | ^[73-76]^ |
|  | Age-related sarcopenia | - Bench research | Level 5 | ^[77]^ |
|  | Age-related macular degeneration | - Cross-sectional study | Level 4 | ^[78]^ |
|  | Age-related hearing loss | - Bench research | Level 5 | ^[79-81]^ |
|  | Skin aging and hair loss | - Cross-sectional study | Level 4 | ^[82]^ |
|  | Infections and autoimmune disease | - Cross-sectional study | Level 4 | ^[83-85]^ |
|  |  | - Bench research | Level 5 | ^[86,87]^ |

**Reference**

[1]. Hildreth KL, Kohrt WM, Moreau KL. Oxidative stress contributes to large elastic arterial stiffening across the stages of the menopausal transition. *Menopause (New York, NY).* 2014;21(6):624-632.

[2]. Muka T, Oliver-Williams C, Kunutsor S, et al. Association of Age at Onset of Menopause and Time Since Onset of Menopause With Cardiovascular Outcomes, Intermediate Vascular Traits, and All-Cause Mortality: A Systematic Review and Meta-analysis. *JAMA Cardiol.* 2016;1(7):767-776.

[3]. Appiah D, Schreiner PJ, Demerath EW, Loehr LR, Chang PP, Folsom AR. Association of Age at Menopause With Incident Heart Failure: A Prospective Cohort Study and Meta-Analysis. *Journal of the American Heart Association.* 2016;5(8).

[4]. Dam V, van der Schouw YT, Onland-Moret NC, et al. Association of menopausal characteristics and risk of coronary heart disease: a pan-European case-cohort analysis. *International journal of epidemiology.* 2019;48(4):1275-1285.

[5]. El Khoudary SR, Wildman RP, Matthews K, Thurston RC, Bromberger JT, Sutton-Tyrrell K. Progression rates of carotid intima-media thickness and adventitial diameter during the menopausal transition. *Menopause (New York, NY).* 2013;20(1):8-14.

[6]. Khan ZA, Janssen I, Mazzarelli JK, et al. Serial Studies in Subclinical Atherosclerosis During Menopausal Transition (from the Study of Women's Health Across the Nation). *The American journal of cardiology.* 2018;122(7):1161-1168.

[7]. Son MK, Lim NK, Lim JY, et al. Difference in blood pressure between early and late menopausal transition was significant in healthy Korean women. *BMC Womens Health.* 2015;15:64.

[8]. Medzikovic L, Aryan L, Eghbali M. Connecting sex differences, estrogen signaling, and microRNAs in cardiac fibrosis. *J Mol Med (Berl).* 2019;97(10):1385-1398.

[9]. Jovanović A. Ageing, gender and cardiac sarcolemmal K(ATP) channels. *J Pharm Pharmacol.* 2006;58(12):1585-1589.

[10]. Xing D, Nozell S, Chen YF, Hage F, Oparil S. Estrogen and mechanisms of vascular protection. *Arterioscler Thromb Vasc Biol.* 2009;29(3):289-295.

[11]. Xu X, Wang B, Ren C, et al. Recent Progress in Vascular Aging: Mechanisms and Its Role in Age-related Diseases. *Aging and disease.* 2017;8(4):486-505.

[12]. Wang D, Oparil S, Chen YF, et al. Estrogen treatment abrogates neointima formation in human C-reactive protein transgenic mice. *Arterioscler Thromb Vasc Biol.* 2005;25(10):2094-2099.

[13]. Huque H, Eramudugolla R, Chidiac B, et al. Could Country-Level Factors Explain Sex Differences in Dementia Incidence and Prevalence? A Systematic Review and Meta-Analysis. *Journal of Alzheimer's disease : JAD.* 2023;91(4):1231-1241.

[14]. Ryan J, Scali J, Carrière I, et al. Impact of a premature menopause on cognitive function in later life. *BJOG : an international journal of obstetrics and gynaecology.* 2014;121(13):1729-1739.

[15]. Rocca WA, Lohse CM, Smith CY, Fields JA, Machulda MM, Mielke MM. Association of Premenopausal Bilateral Oophorectomy With Cognitive Performance and Risk of Mild Cognitive Impairment. *JAMA network open.* 2021;4(11):e2131448.

[16]. Bove R, Secor E, Chibnik LB, et al. Age at surgical menopause influences cognitive decline and Alzheimer pathology in older women. *Neurology.* 2014;82(3):222-229.

[17]. Hestiantoro A, Wiwie M, Shadrina A, Ibrahim N, Purba JS. FSH to estradiol ratio can be used as screening method for mild cognitive impairment in postmenopausal women. *Climacteric : the journal of the International Menopause Society.* 2017;20(6):577-582.

[18]. Xi H, Gan J, Liu S, et al. Reproductive factors and cognitive impairment in natural menopausal women: A cross-sectional study. *Frontiers in endocrinology.* 2022;13:893901.

[19]. Grimm A, Mensah-Nyagan AG, Eckert A. Alzheimer, mitochondria and gender. *Neuroscience and biobehavioral reviews.* 2016;67:89-101.

[20]. Amtul Z, Wang L, Westaway D, Rozmahel RF. Neuroprotective mechanism conferred by 17beta-estradiol on the biochemical basis of Alzheimer's disease. *Neuroscience.* 2010;169(2):781-786.

[21]. Kusters CDJ, Paul KC, Duarte Folle A, et al. Increased Menopausal Age Reduces the Risk of Parkinson's Disease: A Mendelian Randomization Approach. *Movement disorders : official journal of the Movement Disorder Society.* 2021;36(10):2264-2272.

[22]. Yadav R, Shukla G, Goyal V, Singh S, Behari M. A case control study of women with Parkinson's disease and their fertility characteristics. *Journal of the neurological sciences.* 2012;319(1-2):135-138.

[23]. Frentzel D, Judanin G, Borozdina O, Klucken J, Winkler J, Schlachetzki JCM. Increase of Reproductive Life Span Delays Age of Onset of Parkinson's Disease. *Frontiers in neurology.* 2017;8:397.

[24]. Nitkowska M, Czyżyk M, Friedman A. Reproductive life characteristics in females affected with Parkinson's disease and in healthy control subjects - a comparative study on Polish population. *Neurologia i neurochirurgia polska.* 2014;48(5):322-327.

[25]. Canonico M, Pesce G, Bonaventure A, et al. Increased Risk of Parkinson's Disease in Women after Bilateral Oophorectomy. *Movement disorders : official journal of the Movement Disorder Society.* 2021;36(7):1696-1700.

[26]. Jurado-Coronel JC, Cabezas R, Ávila Rodríguez MF, Echeverria V, García-Segura LM, Barreto GE. Sex differences in Parkinson's disease: Features on clinical symptoms, treatment outcome, sexual hormones and genetics. *Front Neuroendocrinol.* 2018;50:18-30.

[27]. Li XZ, Sui CY, Chen Q, Zhuang YS, Zhang H, Zhou XP. The effects and mechanism of estrogen on rats with Parkinson's disease in different age groups. *Am J Transl Res.* 2016;8(10):4134-4146.

[28]. Rodriguez-Perez AI, Valenzuela R, Villar-Cheda B, Guerra MJ, Lanciego JL, Labandeira-Garcia JL. Estrogen and angiotensin interaction in the substantia nigra. Relevance to postmenopausal Parkinson's disease. *Exp Neurol.* 2010;224(2):517-526.

[29]. Labandeira-Garcia JL, Rodriguez-Perez AI, Valenzuela R, Costa-Besada MA, Guerra MJ. Menopause and Parkinson's disease. Interaction between estrogens and brain renin-angiotensin system in dopaminergic degeneration. *Frontiers in neuroendocrinology.* 2016;43:44-59.

[30]. Finkelstein JS, Brockwell SE, Mehta V, et al. Bone mineral density changes during the menopause transition in a multiethnic cohort of women. *J Clin Endocrinol Metab.* 2008;93(3):861-868.

[31]. Gopinath V. Osteoporosis. *Med Clin North Am.* 2023;107(2):213-225.

[32]. Curry SJ, Krist AH, Owens DK, et al. Screening for Osteoporosis to Prevent Fractures: US Preventive Services Task Force Recommendation Statement. *Jama.* 2018;319(24):2521-2531.

[33]. Riggs BL, Melton LJ, 3rd. The prevention and treatment of osteoporosis. *N Engl J Med.* 1992;327(9):620-627.

[34]. Iqbal J, Sun L, Zaidi M. Commentary-FSH and bone 2010: evolving evidence. *Eur J Endocrinol.* 2010;163(1):173-176.

[35]. Management of osteoporosis in postmenopausal women: the 2021 position statement of The North American Menopause Society. *Menopause (New York, NY).* 2021;28(9):973-997.

[36]. Epidemiology of osteoporosis and fragility fractures.

[37]. Boyce BF, Xing L. Functions of RANKL/RANK/OPG in bone modeling and remodeling. *Archives of biochemistry and biophysics.* 2008;473(2):139-146.

[38]. Pivonka P, Calvo-Gallego JL, Schmidt S, Martínez-Reina J. Advances in mechanobiological pharmacokinetic-pharmacodynamic models of osteoporosis treatment - Pathways to optimise and exploit existing therapies. *Bone.* 2024;186:117140.

[39]. Qian D, Wang ZF, Cheng YC, Luo R, Ge SW, Xu G. Early Menopause May Associate With a Higher Risk of CKD and All-Cause Mortality in Postmenopausal Women: An Analysis of NHANES, 1999-2014. *Frontiers in medicine.* 2022;9:823835.

[40]. Kattah AG, Smith CY, Gazzuola Rocca L, Grossardt BR, Garovic VD, Rocca WA. CKD in Patients with Bilateral Oophorectomy. *Clin J Am Soc Nephrol.* 2018;13(11):1649-1658.

[41]. Murphy D, McCulloch CE, Lin F, et al. Trends in prevalence of chronic kidney disease in the United States. 2016;165(7):473-481.

[42]. National Institutes of Health %J Bethesda MTNIoH, National Institute of Diabetes, Digestive, Diseases K. US Renal Data System, USRDS 2000 Annual Data Report. 2000.

[43]. Ahn SY, Choi YJ, Kim J, Ko GJ, Kwon YJ, Han K. The beneficial effects of menopausal hormone therapy on renal survival in postmenopausal Korean women from a nationwide health survey. *Sci Rep.* 2021;11(1):15418.

[44]. Masrouri S, Alijanzadeh D, Amiri M, Azizi F, Hadaegh F. Predictors of decline in kidney function in the general population: a decade of follow-up from the Tehran Lipid and Glucose Study. *Annals of medicine.* 2023;55(1):2216020.

[45]. Neugarten J, Acharya A, Lei J, Silbiger S. Selective estrogen receptor modulators suppress mesangial cell collagen synthesis. *American journal of physiology Renal physiology.* 2000;279(2):F309-318.

[46]. Cevik EC, Erel CT, Ozcivit Erkan IB, et al. Chronic kidney disease and menopausal health: An EMAS clinical guide. *Maturitas.* 2025;192:108145.

[47]. Urbieta-Caceres VH, Syed FA, Lin J, et al. Age-dependent renal cortical microvascular loss in female mice. *Am J Physiol Endocrinol Metab.* 2012;302(8):E979-986.

[48]. Matsuo K, Gualtieri MR, Cahoon SS, et al. Surgical menopause and increased risk of nonalcoholic fatty liver disease in endometrial cancer. *Menopause (New York, NY).* 2016;23(2):189-196.

[49]. Klair JS, Yang JD, Abdelmalek MF, et al. A longer duration of estrogen deficiency increases fibrosis risk among postmenopausal women with nonalcoholic fatty liver disease. *Hepatology (Baltimore, Md).* 2016;64(1):85-91.

[50]. Wang X, Lu Y, Wang E, et al. Hepatic estrogen receptor α improves hepatosteatosis through upregulation of small heterodimer partner. *Journal of hepatology.* 2015;63(1):183-190.

[51]. Gutierrez-Grobe Y, Ponciano-Rodríguez G, Ramos MH, Uribe M, Méndez-Sánchez N. Prevalence of non alcoholic fatty liver disease in premenopausal, posmenopausal and polycystic ovary syndrome women. The role of estrogens. *Annals of hepatology.* 2010;9(4):402-409.

[52]. Yang JD, Abdelmalek MF, Pang H, et al. Gender and menopause impact severity of fibrosis among patients with nonalcoholic steatohepatitis. *Hepatology (Baltimore, Md).* 2014;59(4):1406-1414.

[53]. Salvoza NC, Pablo J. Giraudi, Claudio Tiribelli, and Natalia Rosso. Sex differences in non-alcoholic fatty liver disease: hints for future management of the disease. *Exploration of Medicine.* 2020;1(2):51-74.

[54]. Kur P, Kolasa-Wołosiuk A, Misiakiewicz-Has K, Wiszniewska B. Sex Hormone-Dependent Physiology and Diseases of Liver. *International journal of environmental research and public health.* 2020;17(8).

[55]. Imbert-Fernandez Y, Clem BF, O'Neal J, et al. Estradiol stimulates glucose metabolism via 6-phosphofructo-2-kinase (PFKFB3). *The Journal of biological chemistry.* 2014;289(13):9440-9448.

[56]. Yang M, Ma F, Guan M. Role of Steroid Hormones in the Pathogenesis of Nonalcoholic Fatty Liver Disease. *Metabolites.* 2021;11(5).

[57]. Palmisano BT, Zhu L, Stafford JM. Role of Estrogens in the Regulation of Liver Lipid Metabolism. *Advances in experimental medicine and biology.* 2017;1043:227-256.

[58]. Fukata Y, Yu X, Imachi H, et al. 17β-Estradiol regulates scavenger receptor class BI gene expression via protein kinase C in vascular endothelial cells. *Endocrine.* 2014;46(3):644-650.

[59]. Kireev RA, Tresguerres AC, Garcia C, et al. Hormonal regulation of pro-inflammatory and lipid peroxidation processes in liver of old ovariectomized female rats. *Biogerontology.* 2010;11(2):229-243.

[60]. Pafili K, Paschou SA, Armeni E, Polyzos SA, Goulis DG, Lambrinoudaki I. Non-alcoholic fatty liver disease through the female lifespan: the role of sex hormones. *J Endocrinol Invest.* 2022;45(9):1609-1623.

[61]. Brand JS, van der Schouw YT, Onland-Moret NC, et al. Age at menopause, reproductive life span, and type 2 diabetes risk: results from the EPIC-InterAct study. *Diabetes care.* 2013;36(4):1012-1019.

[62]. LeBlanc ES, Kapphahn K, Hedlin H, et al. Reproductive history and risk of type 2 diabetes mellitus in postmenopausal women: findings from the Women's Health Initiative. *Menopause (New York, NY).* 2017;24(1):64-72.

[63]. Mishra SR, Waller M, Chung HF, Mishra GD. Epidemiological studies of the association between reproductive lifespan characteristics and risk of Type 2 diabetes and hypertension: A systematic review. *Maturitas.* 2022;155:14-23.

[64]. Park SK, Harlow SD, Zheng H, et al. Association between changes in oestradiol and follicle-stimulating hormone levels during the menopausal transition and risk of diabetes. *Diabetic medicine : a journal of the British Diabetic Association.* 2017;34(4):531-538.

[65]. Lambrinoudaki I, Paschou SA, Armeni E, Goulis DG. The interplay between diabetes mellitus and menopause: clinical implications. *Nat Rev Endocrinol.* 2022;18(10):608-622.

[66]. de Mutsert R, Gast K, Widya R, et al. Associations of Abdominal Subcutaneous and Visceral Fat with Insulin Resistance and Secretion Differ Between Men and Women: The Netherlands Epidemiology of Obesity Study. *Metabolic syndrome and related disorders.* 2018;16(1):54-63.

[67]. Hayatbakhsh MR, Najman JM, O'Callaghan MJ, Williams GM, Paydar A, Clavarino A. Association between smoking and respiratory function before and after menopause. *Lung.* 2011;189(1):65-71.

[68]. Tang R, Fraser A, Magnus MC. Female reproductive history in relation to chronic obstructive pulmonary disease and lung function in UK biobank: a prospective population-based cohort study. *BMJ open.* 2019;9(10):e030318.

[69]. Xu X, Jones M, Mishra GD. Age at natural menopause and development of chronic conditions and multimorbidity: results from an Australian prospective cohort. *Human reproduction (Oxford, England).* 2020;35(1):203-211.

[70]. Liang C, Chung HF, Dobson A, Sandin S, Weiderpass E, Mishra GD. Female reproductive histories and the risk of chronic obstructive pulmonary disease. *Thorax.* 2024;79(6):508-514.

[71]. Real FG, Svanes C, Omenaas ER, et al. Lung function, respiratory symptoms, and the menopausal transition. *The Journal of allergy and clinical immunology.* 2008;121(1):72-80.e73.

[72]. Amaral AF, Strachan DP, Gómez Real F, Burney PG, Jarvis DL. Lower lung function associates with cessation of menstruation: UK Biobank data. *The European respiratory journal.* 2016;48(5):1288-1297.

[73]. Herring MJ, Avdalovic MV, Lasley B, Putney LF, Hyde DM. Elderly Female Rhesus Macaques Preserve Lung Alveoli With Estrogen/Progesterone Therapy. *Anatomical record (Hoboken, NJ : 2007).* 2016;299(7):973-978.

[74]. Massaro D, Massaro GD. Estrogen regulates pulmonary alveolar formation, loss, and regeneration in mice. *American journal of physiology Lung cellular and molecular physiology.* 2004;287(6):L1154-1159.

[75]. Massaro D, Massaro GD. Toward therapeutic pulmonary alveolar regeneration in humans. *Proceedings of the American Thoracic Society.* 2006;3(8):709-712.

[76]. Xie B, Chen Q, Dai Z, Jiang C, Chen X. Progesterone (P4) ameliorates cigarette smoke-induced chronic obstructive pulmonary disease (COPD). *Molecular medicine (Cambridge, Mass).* 2024;30(1):123.

[77]. Samad N, Nguyen HH, Scott D, Ebeling PR, Milat F. Musculoskeletal Health in Premature Ovarian Insufficiency. Part One: Muscle. *Semin Reprod Med.* 2020;38(4-05):277-288.

[78]. Elliot S, Catanuto P, Fernandez P, et al. Subtype specific estrogen receptor action protects against changes in MMP-2 activation in mouse retinal pigmented epithelial cells. *Exp Eye Res.* 2008;86(4):653-660.

[79]. Pinaud R, Tremere LA. Control of central auditory processing by a brain-generated oestrogen. *Nat Rev Neurosci.* 2012;13(8):521-527.

[80]. Williamson TT, Ding B, Zhu X, Frisina RD. Hormone replacement therapy attenuates hearing loss: Mechanisms involving estrogen and the IGF-1 pathway. *Aging Cell.* 2019;18(3):e12939.

[81]. Feng ZY, Huang TL, Li XR, et al. 17β-Estradiol promotes angiogenesis of stria vascular in cochlea of C57BL/6J mice. *Eur J Pharmacol.* 2021;913:174642.

[82]. Brincat MP, Baron YM, Galea R. Estrogens and the skin. *Climacteric : the journal of the International Menopause Society.* 2005;8(2):110-123.

[83]. Smith JS, Melendy A, Rana RK, Pimenta JM. Age-specific prevalence of infection with human papillomavirus in females: a global review. *The Journal of adolescent health : official publication of the Society for Adolescent Medicine.* 2008;43(4 Suppl):S5-25, S25.e21-41.

[84]. Andany N, Kennedy VL, Aden M, Loutfy M. Perspectives on menopause and women with HIV. *International journal of women's health.* 2016;8:1-22.

[85]. Desai MK, Brinton RD. Autoimmune Disease in Women: Endocrine Transition and Risk Across the Lifespan. *Frontiers in endocrinology.* 2019;10:265.

[86]. Gameiro CM, Romão F, Castelo-Branco C. Menopause and aging: changes in the immune system--a review. *Maturitas.* 2010;67(4):316-320.

[87]. Gameiro C, Romao F. Changes in the immune system during menopause and aging. *Frontiers in bioscience (Elite edition).* 2010;2(4):1299-1303.
